# INOVATYON/ ENGOT-ov5 study: Randomized phase III international study comparing trabectedin/pegylated liposomal doxorubicin (PLD) followed by platinum at progression *vs* carboplatin/PLD in patients with recurrent ovarian cancer progressing within 6-12 months after last platinum line

**DOI:** 10.1038/s41416-022-02108-7

**Published:** 2023-02-09

**Authors:** N. Colombo, A. Gadducci, J. Sehouli, E. Rulli, J. Mäenpää, C. Sessa, A. Montes, N. B. Ottevanger, R. Berger, I. Vergote, M. D’Incalci, C. Churruca Galaz, R. Chekerov, G. B. Nyvang, S. Riniker, R. Herbertson, R. Fossati, M. P. Barretina-Ginesta, M. Deryal, M. R. Mirza, E. Biagioli, M. Iglesias, G. Funari, M. Romeo, G. Tasca, B. Pardo, G. Tognon, M. J. Rubio-Pérez, A. DeCensi, U. De Giorgi, P. Zola, P. Benedetti Panici, M. Aglietta, V. Arcangeli, C. Zamagni, A. Bologna, A. Westermann, V. Heinzelmann-Schwarz, I. Tsibulak, P. Wimberger, A. Poveda, INOVATYON study group, Nicoletta Colombo, Angiolo Gadducci, Eliana Rulli, Elena Biagioli, Roldano Fossati, Giuseppe Funari, Luciano Carlucci, Davide Poli, Maria Clara Caudana, Giulia Tasca, Maria Ornella Nicoletto, Germana Tognon, Andrea DeCensi, Ugo De Giorgi, Paolo Zola, Dionyssios Katsaros, Pierluigi Benedetti Panici, Innocenza Palaia, Massimo Aglietta, Valentina Arcangeli, Claudio Zamagni, Alessandra Bologna, Alessandro Bertolini, Cinzia Caroti, Milena Bruzzone, Nicoletta Donadello, Gianna Di Costanzo, Alberto Zaniboni, Daniela Surico, Roberta Buosi, Enrico Cortesi, Elena Zafarana, Vittorio Fusco, Laura Zavallone, Teresa Gamucci, Filomena Narducci, Valentina Musacchi, Luciana Babilonti, Annamaria Ferrero, Luigi Cavanna, Roberto Sabbatini, Stefano Tamberi, Maria Rosa Gentili, Grazia Artioli, Antonio Ardizzoia, Alessia Caldara, Zuzana Sirotovà, Clelia Casartelli, Michele Aieta, Saverio Cinieri, Elvira De Marino, Stefania Gori, Francesco Ferraù, Livio Blasi, Massimiliano Alù, Sabino De Placido, Carlo Milandri, Cristina Churruca Galaz, Maria Pilar Barretina-Ginesta, Isabel Bover, Margarita Romeo, Beatriz Pardo, Maria Jesus Rubio-Pèrez, Andrés Poveda, Ana Santaballa, Raúl Márquez, Jesus Alarcon, Cristina Caballero-Diaz, Nuria Ruiz Miravet, Eugenia Ortega, Maria Angels Arcusa Lanza, Silvia Catot Tort, Elena Garcia Martinez, Regina Girones, Yolanda Garcia, Cesar Mendiola, Ana Beatriz Sanchez, Elena Garcia Martinez, Jalid Sehouli, Mustafa Deryal, Pauline Wimberger, Georg Heinrich, Ingo Runnebaum, Fabian Trillsch, Gülten Oskay-Özcelik, Maike de Wit, Eva-Maria Grischke, Dirk Bauerschlag, Florian Heitz, Alexander Mustea, Tanja Fehm, Andrea Heider, Max Dieterich, Martina Groop-Meier, Marco Battista, Achim Woeckel, Ivo Meinhold-Heerlein, Ana Montes, Rebecca Herbertson, Emma Hudson, Rebecca Bowen, Ignace Vergote, Lionel D’Hondt, Peter Vuylsteke, Christof Vulsteke, Petronella-Beatrix Ottevanger, Anneke M. Westermann, Cristiana Sessa, Salome Riniker, Viola Heinzelmann-Schwarz, Roger Von Moos, Elena Kralidis, Michael Mueller, Stefan Aebi, Catrina Uhlmann Nussbaum, Mathias Fehr, Andreas Müller, Christian Taverna, Johanna Mäenpää, Gitte-Bettina Nyvang, Mansoor Raza Mirza, Gunnar B. Kristensen, Anne Gry Bentzen, Bent Fiane, Ulla Puistola, Maarit Anttila, Christian Marth, Regina Berger, Edgar Petru, Christian Schauer, Alexander Reinthaller

**Affiliations:** 1 Gynecologic Cancer Program, European Institute of Oncology IRCCS and University of Milan-Bicocca, Milan, Italy; 2 Clinical and Experimental Medicine, Division of Gynecology and Obstetrics, University of Pisa, Pisa, Italy; 3 North Eastern German Society of Gynecological Oncology (NOGGO) Study group and Gynecology with Center for Oncological Surgery, European Competence Center for Ovarian Cancer, Charité-Berlin University of Medicine, Berlin, Germany; 4 Istituto di Ricerche Farmacologiche Mario Negri IRCCS, Milan, Italy; 5 Nordic Society of Gynecologic Oncology Clinical Trial Unit (NSGO-CTU) & Department of Obstetrics and Gynaecology and Tays Cancer Centre Tampere University and University Hospital, Tampere, Finland; 6 Istituto Oncologico della Svizzera Italiana, IOSI and Swiss Group for Clinical Cancer Research (SAKK), Bellinzona, Switzerland; 7 Guys and St Thomas’ NHS Foundation Trust, London, UK; 8 Medical Oncology, Radboud University Medical Center, Nijmegen, Netherlands; 9 Medical University of Innsbruck, Department for Gynecology and Obstetrics, Innsbruck, Austria, and AGO, Innsbruck, Austria; 10 Gynecologic Oncology, University Hospitals Leuven, Leuven Cancer Institute, and Belgian Gynaecological Oncology Group (BGOG), European Union, Leuven, Belgium; 11 Department of Biomedical Sciences, Humanitas University, Pieve Emanuele, Italy; 12 Hospital de Donosti San Sebastián and Grupo Español de Cáncer de Ovario (GEICO), de Donosti San, Spain; 13 Nordic Society of Gynecologic Oncology Clinical Trial Unit (NSGO-CTU) & Department of Oncology, Odense University Hospital, Odense, Denmark; 14 Oncology, Kantonsspital St. Gallen and Swiss Group for Clinical Cancer Research (SAKK), St. Gallen, Switzerland; 15 Brighton and Sussex University Hospitals NHS Trust, Brighton, UK; 16 Institut Catalá de Oncología de Girona and Grupo Español de Cáncer de Ovario (GEICO), de Donosti San, Spain; 17 North Eastern German Society of Gynecological Oncology (NOGGO) Study group and CaritasKlinikum St. Theresia—Saarbruecken, Saarbruecken, Germany; 18 Nordic Society of Gynecologic Oncology Clinical Trial Unit (NSGO-CTU) & Department of Cancer Treatment, Copenhagen University Hospital, Copenhagen, Denmark; 19 Hospital Son Llázter and Grupo Español de Cáncer de Ovario (GEICO), Palma, Spain; 20 Institut Catalá de Oncología de Badalona and Grupo Español de Cáncer de Ovario (GEICO), de Donosti San, Spain; 21 Oncologia 2, Istituto Oncologico Veneto, Padua, Italy; 22 Institut Catalá de Oncología de Hospitalet and Grupo Español de Cáncer de Ovario (GEICO), Barcelona, Spain; 23 Obstetrics and Gynaecology, ASST Spedali Civili-Università degli Studi di Brescia, Brescia, Italy; 24 Hospital Reina Sofía Córdoba and Grupo Español de Cáncer de Ovario (GEICO), de Donosti San, Spain; 25 S.C. Oncologia Medica, E.O. Ospedali Galliera, Genoa, Italy; 26 IRCCS Istituto Romagnolo per lo Studio dei Tumori (IRST) “Dino Amadori”, Meldola, Italy; 27 AOU Città della Salute e della Scienza - OIRM S. Anna, Torino, Italy; 28 AOU Policlinico Umberto I di Roma, Rome, Italy; 29 Istituto di Candiolo, FPO-IRCCS and University of Torino, Torino, Italy; 30 Ospedale Infermi, Rimini, Italy; 31 IRCCS Azienda Ospedaliero-Universitaria di Bologna, Bologna, Italy; 32 Azienda Unità Sanitaria Locale di Reggio Emilia IRCCS, Reggio Emilia, Italy; 33 Amsterdam UMC, Amsterdam, Netherlands; 34 Universitätsspital Basel, USB andSwiss Group for Clinical Cancer Research (SAKK), Basel, Switzerland; 35 North Eastern German Society of Gynecological Oncology (NOGGO) Study group and Department of Gynecology and Obstetrics, Technische Universität Dresden, Fetscherstr, Dresden, Germany; 36 National Center for Tumor Diseases (NCT/UCC), Dresden, Germany; 37 German Cancer Research Center (DKFZ), Heidelberg, Germany; 38 Faculty of Medicine and University Hospital Carl Gustav Carus, Technische Universität Dresden, Dresden, Germany; 39 Helmholtz-Zentrum Dresden - Rossendorf (HZDR), Dresden, Germany; 40 Initia Oncologia and Grupo Español de Cáncer de Ovario (GEICO), Valencia, Spain; 41 Ospedale Civile, Sondrio, Italy; 42 IRCCS Ospedale Policlinico San Martino, Genova, Italy; 43 Ospedale Del Ponte, Varese, Italy; 44 Azienda Ospedaliera S. Croce e Carle, Cuneo, Italy; 45 Fondazione Poliambulanza, Brescia, Italy; 46 AOU Maggiore della Carità, Novara, Italy; 47 Policlinico Umberto I, Università di Roma “La Sapienza”, Roma, Italy; 48 Nuovo Ospedale di Prato S. Stefano, Prato, Italy; 49 Azienda Ospedaliera SS Antonio e Biagio e Cesare Arrigo, Alessandria, Italy; 50 Ospedale Degli Infermi, Biella, Italy; 51 Ospedale SS Trinità, Sora, Italy; 52 IRCCS Policlinico S. Matteo, Pavia, Italy; 53 AO Ordine Mauriziano, Torino, Italy; 54 Ospedale Guglielmo da Saliceto, Piacenza, Italy; 55 Azienda Ospedaliero Universitaria Policlinico di Modena, Modena, Italy; 56 Ospedale di Faenza, Faenza, Italy; 57 Ospedale Umberto I, Lugo, Italy; 58 U.L.S.S. 13 Mirano, Dolo, Noale, Italy; 59 AO della Provincia di Lecco, Ospedale Alessandro Manzoni, Lecco, Italy; 60 Ospedale di Santa Chiara, Trento, Italy; 61 Ospedale Regionale Umberto Parini, Aosta, Italy; 62 Ospedale Valduce, Como, Italy; 63 Istituto di Ricovero e Cura a Carattere Scientifico—Centro Regionale Oncologico Basilicata, Rionero in Vulture, Italy; 64 P.O. “A.Perrino” ASL Brindisi, Brindisi, Italy; 65 ASL VC Ospedale S. Andrea, Vercelli, Italy; 66 Sacro Cuore Don Calabria, Negrar, Italy; 67 Ospedale S. Vincenzo, Taormina, Italy; 68 ARNAS Civico, Palermo, Italy; 69 Università degli Studi di Napoli Federico II, Napoli, Italy; 70 Ospedale San Giuseppe - Azienda, USL11 Empoli, Italy; 71 Hospital La Fe, Valencia, Spain; 72 MD Anderson Cancer Center, Madrid, Spain; 73 Hospital Son Espases - Palma de Mallorca, Mallorca, Spain; 74 Hospital General Universitario de Valencia, Valencia, Spain; 75 Consorcio Hospitalario Provincial de Castellon, Castelló de la Plana, Spain; 76 H.U. Arnau de Vilanova, Lleida, Spain; 77 Consorci Sanitari De Terrassa, Terrassa, Spain; 78 Althaia, Manresa, Spain; 79 Hospital Universitario J.M. Morales Meseguer, Murcia, Spain; 80 Hospital Lluis Alcanyis Xativa, Valencia, Spain; 81 Corporacion Sanitaria y Universitaria Parc Tauli, Sabadell, Spain; 82 Hospital Universitario 12 de Octubre, Madrid, Spain; 83 Hospital General Universitario de Elche, Alicante, Spain; 84 Hospital Clinico Universitario Virgen De La Arrixaca, Murcia, Spain; 85 Schwerpunktpraxis für Gynäkologische Onkologie, Fürstenwalde, Germany; 86 Universitaetsklinikum Jena, Jena, Germany; 87 University Medical Center Hamburg, Hamburg, Germany; 88 Praxisklinik Krebsheilkunde für Frauen, Berlin, Germany; 89 Vivantes Netzwerk für Gesundheit GmbH, Berlin, Germany; 90 Universitäts - Frauenklinik, Tübingen, Germany; 91 Universitaetsklinikum SH Campus Kiel, Kiel, Germany; 92 Kliniken Essen Mitte Evang. Huyssens Stiftung, Essen, Germany; 93 Universitatsmedizin Greifswald, Greifswald, Germany; 94 Universitatsfrauenklinik Dusseldorf, Dusseldorf, Germany; 95 Klinikum Leverkusen gGmbH, Leverkusen, Germany; 96 UFK am Klinikum Suedstadt Rostock, Rostock, Germany; 97 Studienzentrum Onkologie, Ravensburg, Germany; 98 Universitätsklinikum der Johannes-Gutenberg-Universität Mainz, Mainz, Germany; 99 Obstetrics and Gynaecology - Universitatsklinikum Wuerzburg, Wuerzburg, Germany; 100 University Hospital Aachen, Aachen, Germany; 101 Velindre Cancer Center - Whitchurch Cardiff, Cardiff, UK; 102 Royal United Hospital Bath, Bath, UK; 103 CHU Dinant Godinne / UCL Namur, Yvoir, Belgium; 104 CMSE Clinique et Maternité Sainte-Elisabeth, Namur, Belgium; 105 AZ Maria Middelares, Gent, Belgium; 106 Kantonsspital Graubünden, Chur, Switzerland; 107 Kantonsspital Aarau, Aarau, Switzerland; 108 Universitätsklinik für Frauenheilkunde, Universitätsklinik für Onkologische Medizin - Inselspital, Bern, Switzerland; 109 Luzerner Kantonsspital, Kantonsspital, Switzerland; 110 Kantonsspital Olten, Olten, Switzerland; 111 Kantonsspital Frauenfeld, Frauenfeld, Switzerland; 112 Kantonsspital, Winterthur, Switzerland; 113 Kantonsspital, Münsterlingen, Switzerland; 114 Radium Hospitalet Oslo University Hospital, Oslo, Norway; 115 University Hospital of North Norway, Tromsoe, Norway; 116 Stavanger University Hospital, Stavanger, Norway; 117 Oulu University Hospital, Oulu, Finland; 118 Kuopio University Hospital, Kuopio, Finland; 119 Medizinische Universitat Graz, Graz, Austria; 120 Krankenhaus Der Barmherzigen Brueder, Graz, Austria; 121 Univ. Klinik Frauenheilkunde AKH, Wien, Austria

**Keywords:** Ovarian cancer, Chemotherapy

## Abstract

**Background:**

This trial investigated the hypothesis that the treatment with trabectedin/PLD (TP) to extend the platinum-free interval (TFIp) can improve overall survival (OS) in patients with recurrent ovarian cancer (OC).

**Methods:**

Patients with OC (up to two previous platinum-based lines), with a TFIp of 6–12 months, were randomised to receive carboplatin/PLD (CP) or TP followed by platinum therapy at relapse. The primary endpoint was OS (HR: 0.75).

**Results:**

The study enrolled 617 patients. The median TFIp was 8.3 months and 30.3% of patients had received two previous platinum lines. 74% and 73.9% of patients, respectively, received a subsequent therapy (ST) in the CP and TP arm; in the latter TP arm 87.2% of ST was platinum-based, as per protocol. The median OS was 21.4 for CP and 21.9 months for TP (HR 1.13; 95% CI: 0.94–1.35; *p* = 0.197). Grade 3–5 adverse reactions occurred in 37.1% of patients in the CP arm and 69.7% of patients in the TP arm, and the most frequent were neutropenia (22.8% CP, 39.5% TP), gastrointestinal (7.1% CP, 17.4% TP), hepatic (0.7% CP, 19.1% TP).

**Conclusions:**

This study did not meet the primary endpoint. CP combination remains the standard for patients with recurrent OC and a 6–12 months TFIp; TP is an effective treatment in patients suffering from persistent platinum toxicities.

**Clinical trial registration:**

ClinicalTrials.gov, number NCT01379989.

## Introduction

Ovarian carcinoma (OC) accounts for only 2.3% of all new cancer cases in females but it is the fifth leading cause of all cancer-related deaths [1]. Such a burden appears mainly due to the lack of an effective screening program for OC, so most patients present with advanced disease at diagnosis. Despite improvements in surgery and medical therapy many patients experiencing relapses. When patients face relapse after front-line therapies, the time elapsed since last platinum chemotherapy forms a graduated continuum of the probability of response to further chemotherapy. Platinum is considered the most active drug in OC and the platinum-free interval—currently referred to the “treatment-free interval from last platinum dose” (TFIp) [2]—has been considered the main factor to predict the duration of response when platinum is re-administered in subsequent lines. For this reason since the mid90s’ clinicians have explored the option of prolonging the TFIp with platinum-free regimens to boost the activity of platinum rechallenge [3, 4].

We designed this phase III randomized trial to compare a doublet with platinum, i.e. carboplatin and pegylated liposomal doxorubicin-PLD (carboplatin/PLD) with a doublet without platinum, i.e. trabectedin and PLD (trabectedin/PLD) followed by platinum rechallenge at relapse, hypothesizing that this second option might offer an overall survival (OS) benefit.

These two chemotherapy regimens have never been directly compared but had shown an interesting efficacy and toxicity profile in previous phase III randomized trials in the recurrent setting [5, 6]. In fact in the non-inferiority CALYPSO study, the carboplatin/PLD regimen gave a better risk-benefit profile than carboplatin/paclitaxel in the subset of patients with TFIp 6–12 months [7] and OVA-301 trial demonstrated the superiority of trabectedin/PLD over PLD alone in terms of PFS in the overall population and in terms of OS in the subgroup with TFIp 6–12 months [8].

Because of these results the INOVATYON trial was run in patients with recurrent OC and a TFIp 6–12 months; this patient population was deemed “partially platinum sensitive” when the trial was designed and represents about 23% of all OC relapses. This population was considered an approriate clinical setting to explore the hypothesis of increasing platinum efficacy by artificially prolonging the TFIp.

## Methods

INOVATYON was an open-label, international, parallel-group, randomised phase 3 trial conducted at 117 centres in 11 European countries. The trial was overseen by an independent data monitoring committee (IDMC) and a steering committee. The trial was done in accordance with the Declaration of Helsinki and the International Conference on Harmonisation (ICH) E6 Guidelines for Good Clinical Practice. The trial was approved in each country by the competent authority (CA), a central independent ethics committee and by the independent ethics committees at each trial site. All patients provided written informed consent to participate. The study was performed according to the ENGOT Model A [9]. Patients were randomly assigned (with a 1:1 ratio) to the two treatment regimens, by a biased-coin minimisation procedure and according to the following factors: centre, line of chemotherapy (2nd vs. 3rd), measurable disease (yes or no) and previous anthracycline-based chemotherapy (yes or no). An electronic system integrated into the eCRF that automated the random assignments to the treatment groups was used.

### Patients

Eligible women (aged 18 years or over) had epithelial ovarian, epithelial fallopian tube cancer or primary peritoneal cancer; they had received up to two previous platinum-based chemotherapy regimens, of which at least one must have contained a taxane and had relapsed between 6 and 12 months after the last dose of the platinum-based chemotherapy; they had Eastern Cooperative Oncology Group (ECOG) performance status ≤2 and adequate organ function, with measurable or evaluable disease confirmed by radiological imaging.

### Treatments and procedures

Patients received intravenous PLD 30 mg/m^2^ followed by carboplatin area under the curve (AUC) 5 in a 4-week schedule, or intravenous PLD 30 mg/m^2^ followed by trabectedin 1.1 mg/m^2^ in a 3-week schedule. Primary prophylactic intravenous 20 mg dexamethasone 30 min before the PLD infusion was mandatory for all patients randomised to trabectedin. In both arms, the recommended number of treatment cycles was six, although patients with clinical benefits could continue therapy beyond cycle 6 at the discretion of the investigator. In the trabectedin/PLD group, subsequent platinum rechallenge at disease progression was mandatory unless the patient refused it or the general condition did not allow it.

Dose reductions and interruptions were permitted according to criteria based on haematological and non-haematological toxicities reported in the study protocol. A maximum of two dose reductions was allowed, regardless of the type of toxicity. Treatment was permanently discontinued for any patient who required a third dose reduction.

Patients underwent tumour assessments according to RECIST version 1.1 [10] by CT or MRI tumour imaging and CA-125 serum levels at 12 and 24 weeks. After that, pelvic examination and CA-125 levels were done every 12 weeks for the first two years and every 6 months thereafter, until the loss of follow-up, evidence of progression disease or death. Additional radiological disease assessments were done as clinically indicated, to confirm disease progression.

During the trial, health-related quality of life (HRQoL) assessments were done twice: at screening (before randomisation) and within 4 weeks from the end of the sixth cycle or at the time of progression, whichever came first. HRQoL was assessed with two validated questionnaires: the European Organization for Research and Treatment of Cancer Core Quality of Life Questionnaire C30 (EORTC QLQ-C30) [11] and the EORTC QLQ Ovarian Cancer Module 28 (OV28) [12]. Subscale scores range from 0 to 100. For functional scales and global health status, a higher score indicates a better status and for symptom scales, a higher score indicates a worse burden of symptoms. A 10-point difference in the QoL score was considered clinically meaningful. Compliance was measured as the proportion of patients on treatment submitting adequate data as per EORTC QLQ-C30 and EORTC QLQ-OV28 scoring guidelines.

Adverse events were graded according to the National Cancer Institute Common Terminology Criteria for Adverse Events (NCI-CTCAE) version 4.0 and coded using the MedDRA, v.11. Adverse events were collected from informed consent signature until 30 days after the last dose of the drug or until the start of a new antitumor therapy, whichever came first.

### Outcomes

The primary endpoint was OS which was measured from the date of randomisation up to the date of death due to any cause or, for living patients, the date of the last contact.

Secondary endpoints included progression-free survival (PFS), PFS after subsequent therapy (PFS-ST), PFS at second progression (PFS2), safety profile and HRQoL. PFS was measured from the date of randomisation to the date of documented PD or death due to any cause, whichever came first. If a patient received further antitumor therapy before PD, PFS was censored on the date of this anti-tumour therapy. The objective clinical progression that did not require radiological confirmation was peritoneal carcinomatosis with increasing bowel dysfunction, increased ascites requiring drainage, emerging surgical procedures due to bowel obstruction. PFS-ST was defined as the time from the first dose of subsequent treatment (ST) until progression or death due to any cause and analysed on patients receiving a ST after INOVATYON. PFS2, which was not planned in the original protocol but recommended by the IDMC, was taken as the time from the date of randomisation until the second progression or death due to any cause and analysed on randomised patients.

Safety endpoints were: for each drug-related adverse event, the maximum grade experienced by each patient; for each drug-related adverse event, patients experiencing grade 3–4 events. If the same drug-related adverse event occurred two or more times in the same patient, this was counted as a single event and the worst grade was considered.

Type, frequency and nature of serious adverse events (SAEs) and serious adverse drug reactions (SADRs) were also described.

### Statistical analysis

This trial was designed as a superiority study to assess OS in patients receiving the trabectedin/PLD doublet compared with those receiving the reference carboplatin/PLD doublet. The primary hypothesis was that median OS would range between 18 and 24 months in the reference arm on the basis of previously published results and that the trabectedin/PLD doublet would improve median OS by 6–8 months (Hazard Ratio [HR] = 0.75). Assuming a one-sided α of 0.025 and β 0.15 and loss to follow-up 5%, it was calculated that 442 events were needed. Considering an accrual period of 42 months and a follow-up of about 30 months, the trial was planned to recruit 588 patients.

A one-sided α test was chosen because the positive effect of TFIp with trabectedin/PLD was deemed probable, and we were not interested in proving the superiority of the standard treatment.

We compared survival outcomes between treatment groups using a log-rank test. We estimated the HR and 95% CI for survival outcomes for treatment comparisons using a Cox regression model adjusted for the randomisation stratification factors. We used the Kaplan–Meier method to estimate the median PFS and OS for the two treatment groups.

Differences between arms in adverse events were tested with a χ2 test for trend.

For each treatment group, the mean difference between the two time-points of the EORTC QLQ-C30 and EORTC QLQ-OV28 subscales is presented and differences between arms were tested by means *t*-test.

Efficacy analyses were done for patients randomly assigned in the trial according to their original treatment assignment and with no major violations (i.e. intention-to-treat [ITT] population). Safety analyses were done for patients who received at least one chemotherapy cycle.

The IDMC reviewed unblinded safety data on a periodic basis, the results of the futility interim analysis and efficacy interim analysis. The Sponsor trial management staff, clinicians and funder were blinded to the efficacy results until the final analysis of OS.

Statistical analyses were done in SAS (version 9.4).

The study is registered with ClinicalTrials.gov, number NCT01379989 and European Clinical Trials database, EudraCT 2010-022949-17.

## Results

The first patient was randomised in December 2011 but a month later, the study had to be put on temporary hold due to the worldwide shortage of PLD. Patient’s accrual recommenced on 29 November 2013 and by 18 September 2017, 617 patients (306 patients in the carboplatin/PLD and 311 in the trabectedin/PLD group) were randomised from 117 European centres.

A planned futility analysis of the primary endpoint (OS) was done after ~100 events in March 2017. The planned second interim analysis to test superiority was done in September 2018 after two-thirds of the death events, with the significance determined by the observed number of events and alpha spending function defined by the O’Brien-Fleming boundary. In both analyses, the trial was not stopped early for futility or efficacy.

**Fig. 1:**
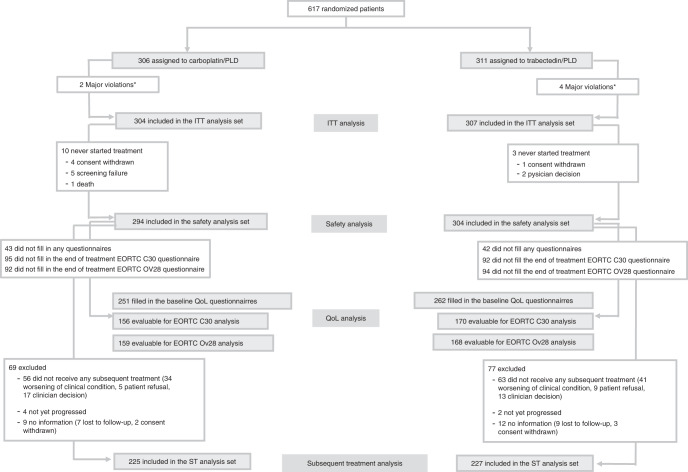
Consort diagram. *Informed consent form not signed/ consent to the procession of personal data not signed; ITT intention to treat, QoL quality of life, ST subsequent treatment, PLD pegylated liposomial doxorubicin.

Six patients were excluded for major violations and 611 were included in the ITT population. Figure 1 depicts the Consort diagram.

The median age was 64.0 years (Q1–Q3:55.0–71.0) and 517 patients (84.6%) had the serous histological type.

About two-thirds of the patients in INOVATYON had received only one previous chemotherapy line, the remaining having received two. The median TFIp since the last platinum-based therapy was 8.3 months. Baseline characteristics were generally balanced between arms and are shown in Table 1.

**Table 1:** Baseline characteristics—ITT analysis set.

	Carboplatin/PLD *N* = 304	Trabectedin/PLD *N* = 307	Overall *N* = 611
Age, years—median (Q1–Q3)	64.0 (55.0–70.0)	63.0 (55.0–71.0)	64.0 (55.0–71.0)
ECOG performance status—*n* (%)			
0	216 (74.2)	208 (68.9)	424 (71.5)
1	70 (24.1)	86 (28.5)	156 (26.3)
2	5 (1.7)	8 (2.6)	13 (2.2)
Missing	13	5	18
Primary site of disease—*n* (%)			
Ovary	277 (91.1)	266 (86.6)	543 (88.9)
Peritoneal	16 (5.3)	25 (8.1)	41 (6.7)
Fallopian	10 (3.3)	16 (5.2)	26 (4.3)
Unknown	1 (0.3)	0 (0.0)	1 (0.2)
Initial FIGO stage—*n* (%)			
I	10 (3.3)	9 (2.9)	19 (3.1)
II	9 (3.0)	4 (1.3)	13 (2.1)
IIIA/B	17 (5.6)	26 (8.5)	43 (7.0)
IIIC	190 (62.5)	178 (58.0)	368 (60.2)
IV	67 (22.0)	72 (23.5)	139 (22.7)
Unknown	11 (3.6)	18 (5.9)	29 (4.7)
Histological grade—*n* (%)			
1	9 (3.0)	6 (2.0)	15 (2.5)
2	25 (8.2)	35 (11.4)	60 (9.8)
3	219 (72.0)	219 (71.3)	438 (71.7)
Unknown	51 (16.8)	47 (15.3)	98 (16.0)
Histological type—*n* (%)			
Serous	253 (83.2)	264 (86.0)	517 (84.6)
Endometroid	10 (3.3)	11 (3.6)	21 (3.4)
Other	34 (11.2)	18 (5.9)	52 (8.5)
Unknown	7 (2.3)	14 (4.6)	21 (3.4)
Presence of measurable disease at study entry—*n* (%)	217 (71.4)	221 (72.0)	438 (71.7)
Size of residual disease after initial surgery—*n* (%)			
≤1 cm	154 (50.7)	162 (52.8)	316 (51.7)
>1 cm	77 (25.3)	69 (22.5)	146 (23.9)
No primary sugery	4 (1.3)	4 (1.3)	8 (1.3)
Unknown	69 (22.7)	72 (23.5)	141 (23.1)
Germline BRCA1 mutational status—*n* (%)			
Mutated	18 (6.0)	29 (9.5)	47 (7.7)
Unknown	115 (38.1)	129 (42.2)	244 (40.1)
Wild-type	169 (56.0)	148 (48.4)	317 (52.1)
Missing	2	1	3
Germline BRCA 2 mutational status—*n* (%)			
Mutated	13 (4.3)	13 (4.2)	26 (4.3)
Unknown	119 (39.4)	131 (42.8)	250 (41.1)
Wild-type	170 (56.3)	162 (52.9)	332 (54.6)
Missing	2	1	3
Germline BRCA mutational status—*n* (%)			
Mutated	30 (9.9)	41 (13.4)	71 (11.7)
Unknown	116 (38.4)	131 (42.8)	247 (40.6)
Wild-type	156 (51.7)	134 (43.8)	290 (47.7)
Missing	2	1	3
Number of prior lines—*n* (%)			
0	1 (0.3)	0 (0.0)	1 (0.2)
1	212 (69.7)	213 (69.4)	425 (69.6)
2	91 (29.9)	94 (30.6)	185 (30.3)
Previous anthracycline-based chemotherapy—*n* (%)	27 (8.9)	28 (9.1)	55 (9.0)
Last prior chemotherapy—type—*n* (%)			
Combination with platinum	216 (71.3)	216 (70.4)	432 (70.8)
Combination with platinum and bevacizumab	74 (24.4)	81 (26.4)	155 (25.4)
Monotherapy with platinum	12 (4.0)	9 (2.9)	21 (3.4)
Other without platinum	1 (0.3)	1 (0.3)	2 (0.3)
Maintenance therapy following last prior chemotherapy—*n* (%)	110 (36.7)	122 (39.9)	232 (38.3)
Maintenance therapy—*n* (%)			
Bevacizumab	99 (90.0)	106 (86.9)	205 (88.4)
Olaparib	6 (5.5)	10 (8.2)	16 (6.9)
Other	5 (4.5)	6 (4.9)	11 (4.7)
Last treatment-free interval from platinum, months—median (Q1–Q3)	8.4 (6.9–9.9)	8.3 (7.0–9.9)	8.3 (7.0–9.9)
Surgery after last progression—*n* (%)	18 (5.9)	26 (8.5)	44 (7.2)
For patients who underwent surgery after last progression: all macroscopic disease debulked—*n* (%)	10 (55.6)	18 (69.2)	28 (63.6)

*Q1–Q3* first–third quartile, *PLD* pegylated liposomial doxorubicin, *SD* standard deviation, *Min–Max* minimum–maximum values.

Thirteen patients did not start INOVATYON treatment (10 in the carboplatin/PLD and 3 in the trabectedin/PLD group). Groups received a median of six cycles. Treatment was discontinued in six cycles in 86 (28.3%) patients in the carboplatin/PLD group and 140 (45.6%) in the trabectedin/PLD group (Table s1).

**Fig. 2:**
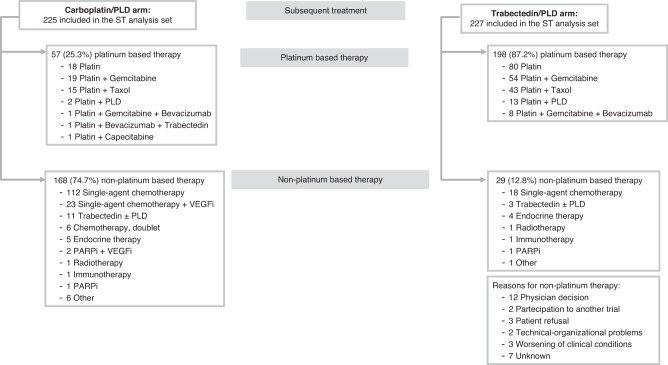
Subsequent treatments. ST subsequent treatment, VEGFi vascular endothelial growth factor inhibitor, PARPi poly ADP-ribose polymerase inhibitors, PLD pegylated liposomial doxorubicin.

In the ITT population 225 (74.0%) of the 304 patients in the carboplatin/PLD group and 227 (73.9%) of the 307 in the trabectedin/PLD arm received further lines of chemotherapy after the study treatment, at disease progression. In the trabectedin/PLD group, 198 (87.2%) of 227 patients received platinum rechallenge as required by the INOVATYON protocol (Fig. 2).

**Fig. 3:**
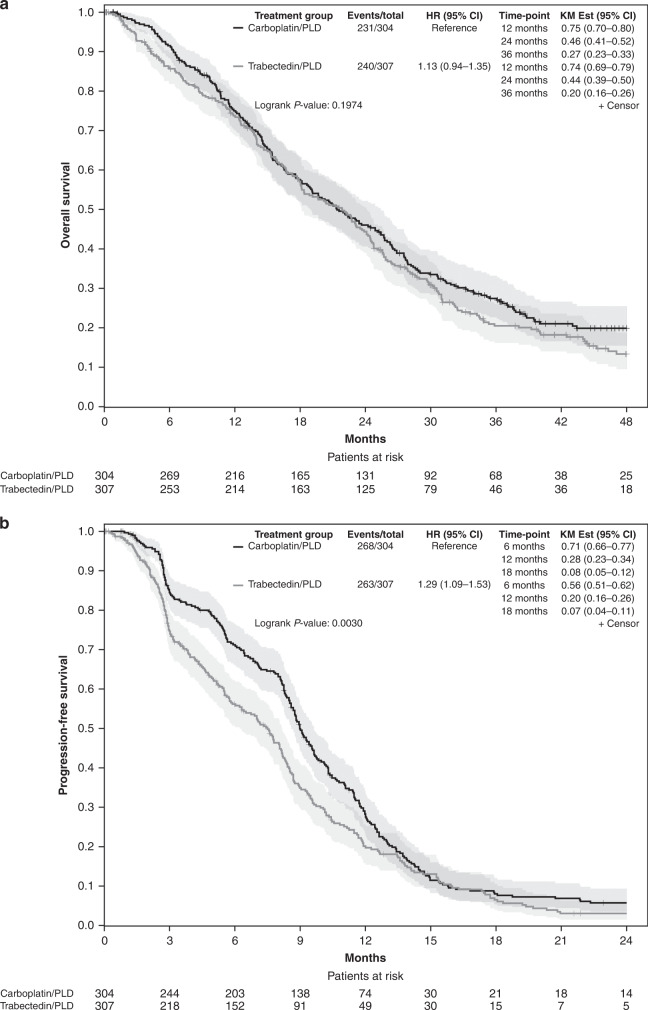
Kaplan-Meier curves. **a** Overall survival; **b** Progression-free survival; CI confidence interval, HR hazard ratio, KM est Kaplan-Meier estimation.

Two-hundred and thirty-one patients (76.0%) in the carboplatin/PLD group and 240 (78.2%) patients in the trabectedin/PLD died. Median overall survival was 21.4 months (95% CI 19.0–25.4) with carboplatin/PLD and 21.9 months (95% CI 18.1–24.1) with trabectedin/PLD (HR 1.13 [95% CI 0.94–1.35]; *p* = 0.197; Log-rank *p* = 0.197; test for proportional hazard *p* = 0.410; Fig. 3a).

Multivariable analysis confirmed the univariate result (Table s2).

Subgroup analyses of OS were consistent with the overall result (Fig. s1). A possible, though not statistically significant, benefit of trabectedin/PLD was seen in patients who had had two 2 prior lines (HR 0.87 95% CI 0.62–1.22), while in patients who had received only one previous line, a statistically significant benefit in favour of carboplatin/PLD was observed (HR 1.25 95% CI 1.01–1.55). In none of the subgroups analysed a significant benefit of trabectedin/PLD over carboplatin/PLD was observed.

After a median follow-up of 45.6 months (first quartile [Q1]–third quartile [Q3]: 36.5–53.5), 268 (88.2%) of the 304 patients in the carboplatin/PLD group and 263 (85.7%) of the 307 in the trabectedin/PLD had progression or had died. Median PFS was 9.0 months (95% CI 8.6–9.6) in the carboplatin/PLD group and 7.5 months (95% CI 6.2–8.2) in the trabectedin/PLD group (HR 1.29 [95% CI 1.09–1.53]; *p* = 0.003; Log-rank *p* = 0.003; Fig. 3b). Since there was clear evidence of non-proportional hazards (test for proportional hazard *p* = 0.001) we calculated the difference in the restricted mean survival time (difference: −1.58 months 95% CI: −2.52 to −0.65, *p* = 0.0009 truncation time: 24 months).

Out of 452 patients who received further therapy after the study treatment, 424 (93.8%) progressed or died. A possible positive impact of trabectedin/PLD treatment on PFS-ST was observed, though not statistically significant (HR 0.87; 95% CI 0.72–1.06; *p* = 0.161). When PFS2 was calculated, the gain in PFS-ST disappeared (PFS2: HR 1.13 95% CI 0.96–1.34; *p* = 0.139). The Kaplan–Meier curves of PFS-ST and PFS2 are depicted in Fig. s2 and Fig. s3.

In the safety population, at least one adverse event of any grade during treatment was reported in 260 (88.4%) of 294 patients in the carboplatin/PLD group and 287 (94.4%) of the 304 patients with trabectedin/PLD. In the former group, 140 (47.6%) of 294 patients had at least one grade 3 or higher adverse event compared with 239 (78.6%) out of 304 patients in the latter group (*p* < 0.001).

At least one drug-related grade 3–5 adverse event (as reported by the investigator) was reported in 109 (37.1%) of the 294 patients in the carboplatin/PLD group and 212 (69.7%) of the 304 in the trabectedin/PLD group. Table 2 lists the frequency of grade 3–4 drug-related adverse events occurring in at least 5% of patients and the drug-related adverse events of grade 5 or events of particular interest regardless of their frequency. All drug-related adverse events are reported in Table s3.

**Table 2:** Maximum grade adverse drug reactions by SOC and PT—adverse drug reactions with at least 5% of frequency for G3–G4 and all G5 reactions regardless of frequency.

Carboplatin/PLD								Trabectedin/PLD							
	G0	G1	G2	G3	G4	G5	G3 + G4 + G5	G0	G1	G2	G3	G4	G5	G3 + G4 + G5	Chi-squared for trend
	*n* (%)	*n* (%)	*n* (%)	*n* (%)	*n* (%)	*n* (%)	*n* (%)	*n* (%)	*n* (%)	*n* (%)	*n* (%)	*n* (%)	*n* (%)	*n* (%)	
Haematological															
Thrombocytopenia	223 (75.9)	21 (7.1)	18 (6.1)	18 (6.1)	14 (4.8)	0 (0.0)	32 (10.9)	263 (86.5)	11 (3.6)	6 (2.0)	14 (4.6)	9 (3.0)	1 (0.3)	24 (7.9)	0.012
Febrile neutropenia	291 (99.0)	0 (0.0)	1 (0.3)	0 (0.0)	2 (0.7)	0 (0.0)	2 (0.7)	289 (95.1)	0 (0.0)	0 (0.0)	9 (3.0)	6 (2.0)	0 (0.0)	15 (4.9)	0.005
Leukopenia	255 (86.7)	7 (2.4)	17 (5.8)	14 (4.8)	1 (0.3)	0 (0.0)	15 (5.1)	259 (85.2)	8 (2.6)	14 (4.6)	14 (4.6)	8 (2.6)	1 (0.3)	23 (7.6)	0.267
Neutropenia	182 (61.9)	10 (3.4)	35 (11.9)	48 (16.3)	19 (6.5)	0 (0.0)	67 (22.8)	145 (47.7)	14 (4.6)	25 (8.2)	64 (21.1)	56 (18.4)	0 (0.0)	120 (39.5)	<0.001
Non-haematological															
Nausea	199 (67.7)	53 (18.0)	35 (11.9)	7 (2.4)	0 (0.0)	0 (0.0)	7 (2.4)	165 (54.3)	59 (19.4)	52 (17.1)	27 (8.9)	1 (0.3)	0 (0.0)	28 (9.2)	<0.001
Vomiting	242 (82.3)	30 (10.2)	17 (5.8)	4 (1.4)	1 (0.3)	0 (0.0)	5 (1.7)	217 (71.4)	43 (14.1)	24 (7.9)	20 (6.6)	0 (0.0)	0 (0.0)	20 (6.6)	0.001
Hepatotoxicity	278 (94.6)	11 (3.7)	3 (1.0)	2 (0.7)	0 (0.0)	0 (0.0)	2 (0.7)	218 (71.7)	9 (3.0)	19 (6.3)	48 (15.8)	10 (3.3)	0 (0.0)	58 (19.1)	<0.001
Sepsis	292 (99.3)	1 (0.3)	0 (0.0)	0 (0.0)	1 (0.3)	0 (0.0)	1 (0.3)	302 (99.3)	0 (0.0)	0 (0.0)	1 (0.3)	0 (0.0)	1 (0.3)	2 (0.7)	0.696
Stomatitis	265 (90.1)	18 (6.1)	9 (3.1)	2 (0.7)	0 (0.0)	0 (0.0)	2 (0.7)	262 (86.2)	16 (5.3)	18 (5.9)	7 (2.3)	1 (0.3)	0 (0.0)	8 (2.6)	0.025
Myelodysplastic syndrome	293 (99.7)	0 (0.0)	0 (0.0)	0 (0.0)	0 (0.0)	1 (0.3)	1 (0.3)	304 (100)	0 (0.0)	0 (0.0)	0 (0.0)	0 (0.0)	0 (0.0)	0 (0.0)	—
Pleural effusion	293 (99.7)	1 (0.3)	0 (0.0)	0 (0.0)	0 (0.0)	0 (0.0)	0 (0.0)	303 (99.7)	0 (0.0)	0 (0.0)	0 (0.0)	0 (0.0)	1 (0.3)	1 (0.3)	—

*PLD* pegylated liposomial doxorubicin, *SOC* System Organ Class (MedDRA), *PT* Preferred Term (MedDRA).

Grade 3–5 drug-related adverse events with an incidence substantially higher in the trabectedin/PLD group than in the carboplatin/PLD group were neutropenia (39.5% vs 22.8%), including febrile neutropenia (4.9% vs 0.7%), gastrointestinal disorders (17.4% vs 7.1%) and hepatotoxicity (19.1% vs 0.7%) (Table 2). Any grade neurotoxicity was 16% for both arms (Table s3), mostly G1 (11.5% vs 10.2%). Twenty patients (6.6%) in carboplatin/PLD group had adverse events leading to treatment discontinuation compared with 38 (12.4%) with trabectedin/ PLD.

Sixty-six patients (22.4%) out of 294 in the carboplatin/PLD group and 130 (42.8%) out of 304 in the trabectedin/PLD group had at least one SAE. Overall, 99 SAEs occurred in the carboplatin/PLD arm and 208 in the trabectedin/PLD arm. Among the 307 SAEs: respectively, 50.5% vs 31.3% were gastrointestinal; 12.1% vs 16.8% were haematological; 8.1% vs. 10.6% were respiratory events in the carboplatin/PLD group vs. the trabectedin/PLD group.

The investigators considered 22 (22.2%) SAEs related to treatment (Serious adverse drug reactions, SADRs) in the carboplatin/PLD group and 91 (43.8%) in the trabectedin/PLD group. Four SADRs had a fatal outcome (1 in the carboplatin/PLD and 3 in the trabectedin/PLD arm). The fatal SADR in the carboplatin/PLD arm was a myelodysplastic syndrome considered related to both drugs. One fatal SADR in the trabectedin/PLD arm was sepsis, considered related only to trabectedin. For the remaining two cases, the disease under study was considered the main cause of death, though some relation with the study drugs cannot be excluded. Moreover, one further fatal event with an unknown cause was recorded in the carboplatin/PLD arm.

HRQoL compliance at baseline was 85.8% overall (with at least one of the two questionnaires completed), 251 of 294 (85.4%) for the carboplatin/PLD group and 262 of 304 (86.2%) for trabectedin/PLD.

HRQOL compliance at the end of the sixth cycle, or progression, considering patients who completed the baseline questionnaire, was: 54.5% overall for EORTC QLQ-C30, 156 (53.1%) for the carboplatin/PLD group and 170 (55.9%) for trabectedin/PLD; 54.7% overall for the EORTC QLQ-OV28 was, 159 (54.1%) for the carboplatin/PLD group and 168 (55.3%) for trabectedin/PLD. Table s4 reports HRQOL score changes.

When comparing HRQOL changes between baseline and after treatment scores, EORTC QLQ-C30 indicated a clinically meaningful (>10 points) negative impact of trabectedin/PLD for global health status, physical functioning, role functioning, social functioning, fatigue, nausea and vomiting, dyspnoea and appetite loss. A clinically relevant worsening of role functioning and fatigue was observed in the carboplatin/PLD arm. For EORTC QLQ-OV28 a clinically significant negative impact of trabectedin/PLD was detected for the scale “Other chemotherapy side-effects”, but no clinically significant effect of carboplatin/PLD was observed in any of the scores.

Looking at the differences between arms in the change scores, a difference of −7.3 points (95% CI −12.4 to −2.2) was detected for global health status, in favour of carboplatin/PLD, and a difference in the increase of fatigue of 8.1 points (95% CI 2.8–13.4), nausea and vomiting (7.8 95% CI 2.5–13.1) and appetite loss (12.6 95% CI 5.9–19.3) was observed, all in favour of carboplatin/PLD. Regarding the QLQ-OV28, a difference of −8.4 points (95% CI −14.3 to −2.5) in the attitude to disease/treatment and a difference of 5.4 (95% CI 0.1–10.7) in hormonal/ menopausal symptoms were observed, again in favour of the control arm.

## Discussion

The trial shows that trabectedin/PLD given to extend the TFIp before platinum rechallenge does not prolong the OS in patients with recurrent OC and a TFIp between 6 and 12 months. OS was similar with both regimens and PFS was better with carboplatin/PLD. Grade 3-4 (5) adverse events were reported more in the trabectedin/PLD group than within the carboplatin/PLD. The trabectedin/PLD regimen was associated with a higher prevalence of haematological, gastrointestinal and hepatic treatment-related adverse events than the carboplatin/PLD regimen.

Trabectedin in association with PLD for the treatment of platinum-sensitive ovarian cancer was approved by EMA in October 2009, but the use of this regimen was not homogeneous across Europe due to the differences in the National Health Systems policies.

This regimen has never been directly compared in a randomised clinical trial with carboplatin/PLD. The hypothesis that prolonging the TFIp by using non-platinum agents could improve response to platinum retreatment was expressed in the ‘90 s but it was never addressed prospectively until the Alvarez Secord’s phase II trial and the MITO-8/ENGOT-ov1 trial were published in 2012 and 2017 [13, 14]. The first study randomised platinum-sensitive patients to docetaxel-carboplatin doublet or docetaxel followed by carboplatin at progression or after six cycles of docetaxel in case of partial response or stable disease. This trial was not comparative, did not attempt to achieve the maximum PFI and ~50% of the patients in the single-agent arm were switched to other treatments before documented disease progression. The MITO-8/ENGOT-ov1 trial compared the efficacy of a non-platinum single agent followed by platinum-based chemotherapy at relapse or the standard platinum-based chemotherapy given first. The trial was conducted in patients with TFIp 6–12 months and failed to show any survival benefit, adopting the sequence single-agent non-platinum-based then platinum-based chemotherapy. The main limitation of this trial was the single-agent strategy used to prolong the TFIp. In fact, the median PFS of the non-platinum-based chemotherapy arm, which in most of the cases was PLD (90.7%), was only 5.0 months, barely more than half the median PFS of the platinum-based arm, which was 9.0 months. We thought that this poor performance could have undermined the catching-up of the platinum rechallenge. The choice of trabectedin as a “non platinum” drug was based on the available molecular pharmacology studies showing important mechanistic differences between this drug and platinum. While platinum drugs bind guanines at N7 position in the major DNA groove forming DNA–DNA and DNA–protein crosslinks, trabectedin binds to guanine at N2 position in the minor DNA groove. Being trabectedin a mono-alkylator cannot form DNA-inter-strand (on the opposite strand) or intra-strand crosslinks between guanines, which are the DNA lesions responsible for the cytotoxicity of platinum drugs. A further difference is the high and low sensitivity of Nucleotide Excision Repair (NER) deficient cells to platinum drugs and to trabectedin, respectively. On the other hand, both platinum drugs and trabectedin are more effective against Homologous Recombination deficient cells, thus suggesting that a certain degree of cross-sensitivity exists.

Although the preclinical models of ovarian cancer do not reproduce the complexity and heterogeneity of human ovarian cancer adequately and their predictivity is not fully demonstrated, nevertheless, in vitro and in vivo studies suggested that the exposure to trabectedin induced a selection of NER-deficient ovarian cancer cells that were very sensitive to platinum drugs, providing a further rational for the choice of trabectedin as a non-platinum drug [15]. These findings were seemingly supported by clinical data from the subgroup analysis of the OVA301 trial that showed a clear advantage in survival for trabectedin/PLD over PLD for patients with TFIp between 6–12 months who had received carboplatin as the first subsequent line [16].

In the post hoc subgroup analyses shown in Fig. s1 carboplatin/PLD tended to perform better in patients who had received one previous platinum-based line of treatment, while in patients receiving two previous lines, the study regimens seems to perform similarly. This observation is in line with the phase II MITO-15 study [17] in heavily pre-treated patients (median four previous lines) treated with trabectedin as a single agent and reporting high response rates of 50% and 33% in patients with TFIp ≥6 months and <6 months, respectively, and with the real-life study NIMES-ROC [18] in platinum-sensitive patients mostly treated with at least 2 previous lines (72.5%), showing median PFS and OS values of 9.46 months and 23.56 months.

A recently published phase III trial by Monk and colleagues [19], comparing the combination of trabectedin/PLD or PLD alone in the third-line setting, i.e. in heavily pre-treated patients, was closed prematurely by the IDMC that recommended its termination because of lack of OS benefit in a pre-planned futility analysis. However, this trial showed a not statistically significant survival benefit for the trabectedin arm in patients with a TFIp of 6–12 months (HR 0.69, 95% CI 0.48–1.01), which was clearly significant in patients who also had a BRCA1/2 mutation (HR 0.37; 95% CI 0.17–0.82). Greater sensitivity to trabectedin in tumours with BRCA mutations was reported long ago in vitro [20, 21] and has recently been confirmed by clinical data [17, 22]. Unfortunately, the results of the Italian randomised MITO-23 phase III trial in BRCA-mutated or BRCAness recurrent OC patients (NCT02903004) were presented at ASCO meeting 2022 and showed that trabectedin as a single agent did not improve OS when compared to physician’s choice chemotherapy [23]. Also, our study did not show any survival benefit in BRCA-mutated patients treated with trabectedin/PLD.

Another specific area where trabectedin might be taken into consideration for tailoring new therapies emerged from the post hoc analysis of the SOLO2/ENGOT ov-21 trial. In fact, this analysis has recently shown a decreased efficacy of subsequent chemotherapy (particularly platinum-based chemotherapy) assessed by time to the second progression, in BRCA1/2 mutated patients having received maintenance olaparib compared to placebo [24].

Our study has some limitations. First, the absence of information on BRCA mutation status in almost 40% of participants, because this was not yet in standard practice during the inclusion time. Secondly, quality of life was assessed only at baseline and at the sixth cycle of chemotherapy or progression, whichever came first, as we tried to obtain an overall description of the therapeutic burden with the minimum number of assessments. However, we might have missed granularity and overlooked elements possibly exerting pronounced effects on quality of life. Moreover, quality of life assessment was not available for all patients and this may lead to bias since data may be missing not at random but actually in relation to patients’ clinical conditions.

This trial was mostly run in the pre-bevacizumab/PARP inhibitors period and these therapeutics are now being integrated into clinical practice and are shaping clinical research. We can only speculate whether trabectedin/ PLD or trabectedin alone might be synergistic with these new medications. A phase II single-arm trial is ongoing in patients with OC on the use of olaparib as maintenance treatment in patients with CR to trabectedin/PLD (NCT03470805). In a randomised phase II clinical trial in patients with TFIp 6–12 months, the combination of trabectedin and bevacizumab showed promising efficacy [25].

This, mature randomised trial did not find any survival advantage compared with standard-of-care platinum-based chemotherapy in the experimental trabectedin/PLD combination. The trabectedin/PLD regimen followed by platinum rechallenge is active in this patient population, showing similar overall survival with respect to platinum-based chemotherapy given first, but it also has higher toxicity and lowers the health-related quality of life. We conclude that a platinum doublet such as carboplatin/PLD combination remains standard practice for recurrent OC patients with a TFIp between 6–12 months; however, trabectedin/PLD can still be considered an option for patients who have received two prior platinum-based lines who show platinum hypersensitivity or may need a longer recovery time from platinum-specific toxicities.

## Supplementary information

Supplementary materials

consort checklist

## Data Availability

Individual participant data that underlie the results reported in this article (text, tables and figures) will be available to be shared, after de-identification and upon request. Data will be available for researchers who provide a methodologically sound proposal and they can be used only to achieve aims in the approved proposal. Proposals should be directed to elena.biagioli@marionegri.it; to gain access, data requestors will need to sign a data access agreement. Data will be available at the following website (“https://zenodo.org/”) as soon as possible but no later than 1 year since the acceptance of this article for publication and for 5 years following article publication.
