# Supplementary material for: INOVATYON/ ENGOT-ov5 study: Randomized phase III international study comparing trabectedin/pegylated liposomal doxorubicin (PLD) followed by platinum at progression vs carboplatin/PLD in patients with recurrent ovarian cancer progressing within 6-12 months after last platinum line
Source: Br J Cancer. 2023 Feb 9;128(8):1503–13. doi: 10.1038/s41416-022-02108-7 (PMC10070417; doi:10.1038/s41416-022-02108-7)
Supplement: Supplementary file 1 — Supplementary materials [file 41416_2022_2108_MOESM1_ESM.pdf]

Table of contents

TABLE S1. TREATMENT COMPLIANCE 2

TABLE S2. THE MULTIVARIABLE ANALYSIS OF OS 3

TABLE S3. MAXIMUM GRADE ADVERSE DRUG REACTIONS BY MEDDRA SYSTEM ORGAN CLASS (SOC) AND PREFERRED TERM (PT) 4

TABLE S4. QUALITY OF LIFE - CHANGE FROM BASELINE 17

FIGURE S1. FOREST PLOT OF OVERALL SURVIVAL BY SUBGROUPS 18

FIGURE S2. KAPLAN-MEIER CURVES FOR PROGRESSION-FREE SURVIVAL AFTER SUBSEQUENT THERAPY (PFS-ST) 19

FIGURE S3. KAPLAN-MEIER CURVES FOR PROGRESSION-FREE SURVIVAL AT THE SECOND PROGRESSION (PFS2) 20

APPENDIX A - INOVATYON STUDY GROUP 21

Table s1. Treatment compliance

	carboplatin/PLD N=304	trabectedin/PLD N=307	OVERALL N=611
**Never started - n (%)**	10 (3.3)	3 (1.0)	13 (2.1)
**Reasons:**			
Consent withdrawn	4 (40.0)	1 (33.3)	5 (38.5)
Screening failure	5 (50.0)	0 (0.0)	5 (38.5)
Physician decision	0 (0.0)	2 (66.7)	2 (15.4)
Death	1 (10.0)	0 (0.0)	1 (7.7)
**Treatment interrupted >=6 cycles - n (%)**	208 (68.4)	164 (53.4)	372 (60.9)
**Reason for the end of treatment:**			
Treatment completed	146 (70.2)	89 (54.3)	235 (63.2)
Disease progression	30 (14.4)	40 (24.4)	70 (18.8)
Physician decision	20 (9.6)	14 (8.5)	34 (9.1)
Unacceptable toxicity	7 (3.4)	12 (7.3)	19 (5.1)
Patient refusal	5 (2.4)	7 (4.3)	12 (3.2)
Intercurrent illness of sufficient severity	0 (0.0)	2 (1.2)	2 (0.5)
**Treatment interrupted < 6 cycles - n (%)**	86 (28.3)	140 (45.6)	226 (37.0)
**Reason for discontinuation:**			
Disease progression	55 (64.0)	70 (50.0)	125 (55.3)
Unacceptable toxicity	13 (15.1)	26 (18.6)	39 (17.3)
Patient refusal	2 (2.3)	15 (10.7)	17 (7.5)
Physician decision	5 (5.8)	8 (5.7)	13 (5.8)
Death	4 (4.7)	6 (4.3)	10 (4.4)
Intercurrent illness of sufficient severity	2 (2.3)	8 (5.7)	10 (4.4)
Consent withdrawn	2 (2.3)	3 (2.1)	5 (2.2)
Lost to follow-up	0 (0.0)	1 (0.7)	1 (0.4)
Screening failure	1 (1.2)	0 (0.0)	1 (0.4)
Other	2 (2.3)	3 (2.1)	5 (2.2)
**Number of cycles**			
Median (Q1 - Q3)	6.0 (6.0-7.5)	6.0 (6.0-9.0)	6.0 (6.0-8.0)
**Maintenance with Olaparib - n (%)**			
No	285 (96.6)	-	285 (96.6)
Yes	10 (3.4)	1 [a]	10 (3.4)
Missing	9	-	9
**Maintenance with other therapy - n (%)**			
No	266 (91.1)	-	266 (91.1)
Yes	26 (8.9)	1 [a]	26 (8.9)
Missing	12	-	12
**Maintenance with other therapy -specify- n (%)**			
Bevacizumab	2 (7.7)	1 (100)	3 (11.1)
Hormonal therapy	5 (19.2)	0 (0.0)	5 (18.5)
Letrozol	1 (3.8)	0 (0.0)	1 (3.7)
Niraparib	11 (42.3)	0 (0.0)	11 (40.7)
Other	7 (26.9)	0 (0.0)	7 (25.9)

Legend: SD: standard deviation. Q1-Q3: First -third quartile. Min-Max: minimum - maximum values.
a : even if the maintenance treatment were not applicable to arm trabectedin/PLD, the site confirmed data

Table s2. The multivariable analysis of OS

	Univariable analysis			Multivariable analysis		
	**HR**	**95% CI**	**P-value**	**HR**	**95% CI**	**P-value**
**Arm (trabectedin/PLD vs. carboplatin/PLD)**	1.13	(0.94-1.35)	0.1974	1.12	(0.93-1.35)	0.2277
**Presence of measurable disease at study entry**	0.96	(0.79-1.18)	0.7099	0.99	(0.80-1.22)	0.9253
**Number of prior treatments (II vs. I)**	0.90	(0.74-1.09)	0.2851	0.89	(0.71-1.11)	0.2891
**Previous anthracycline-based chemotherapy**	1.10	(0.81-1.49)	0.5566	1.00	(0.70-1.43)	0.9886
**Age (1 year increment)**	1.01	(1.00-1.02)	0.0314	1.01	(1.00-1.02)	0.0340
**ECOG performance status (ref. 0)**			<.0001			<.0001
1	1.48	(1.20-1.81)	0.0002	1.46	(1.18-1.79)	0.0003
2	4.34	(2.37-7.94)	<.0001	4.31	(2.30-8.07)	<.0001
**BSA**	0.95	(0.57-1.58)	0.8359			
**Primary site (ref. Ovary)**			0.3464			
Fallopian	1.36	(0.91-2.04)	0.1370			
Peritoneal	1.19	(0.83-1.71)	0.3368			
**Stage at diagnosis (ref. Early)**			0.4952			
Advanced	1.23	(0.87-1.75)	0.2421			
Unknown	1.17	(0.72-1.91)	0.5328			
**Initial FIGO stage**	0.98	(0.92-1.05)	0.5743			
**Hystological Grade (ref. 1)**			0.2381			
2	0.56	(0.30-1.07)	0.0799			
3	0.55	(0.31-0.97)	0.0404			
Unknown	0.57	(0.31-1.05)	0.0698			
**Hystological Type (ref. Serous)**			<.0001			
Endometroid	0.57	(0.31-1.03)	0.0643			
Clear Cell	3.62	(2.18-6.02)	<.0001			
Unspecified	0.65	(0.31-1.37)	0.2555			
Mucinous	2.45	(1.26-4.76)	0.0080			
Mixed Epithelial	0.52	(0.23-1.17)	0.1149			
Transitional	0.42	(0.06-2.98)	0.3837			
Unknown	1.17	(0.71-1.94)	0.5293			
**Size of residual disease after initial surgery (ref. <=1cm)**			0.8013			
>1cm	1.04	(0.83-1.31)	0.7185			
Unknown	1.08	(0.86-1.35)	0.5189			
**Germline BRCA 1 mutational status (ref. Wild-type)**			<.0001			
Mutated	0.68	(0.47-1.01)	0.0532			
Unknown	1.40	(1.16-1.69)	0.0004			
**Germline BRCA 2 mutational status (ref. Wild-type)**			<.0001			
Mutated	0.43	(0.24-0.75)	0.0029			
Unknown	1.38	(1.14-1.66)	0.0007			
**Germline BRCA mutational status (ref. Wild-type)**			<.0001			
Mutated	0.52	(0.37-0.73)	0.0001			
Unknown	1.30	(1.07-1.57)	0.0074			

Table s3. Maximum grade adverse drug reactions by MedDRA system organ class (SOC) and preferred term (PT)

	G0 n (%)	G1 n (%)	G2 n (%)	G3 n (%)	G4 n (%)	G5 n (%)	G3+G4+G5 n (%)	Chi-squared for trend
**Overall**								0.000
Carboplatin/PLD	58 (19.7)	32 (10.9)	95 (32.3)	80 (27.2)	27 (9.2)	2 (0.7)	109 (37.1)	
Trabectedin/PLD	33 (10.9)	13 (4.3)	46 (15.1)	135 (44.4)	74 (24.3)	3 (1.0)	212 (69.7)	
**1. Blood and lymphatic system disorders**								0.000
Carboplatin/PLD	141 (48.0)	15 (5.1)	52 (17.7)	60 (20.4)	26 (8.8)	0 (0.0)	86 (29.3)	
Trabectedin/PLD	114 (37.5)	24 (7.9)	28 (9.2)	76 (25.0)	61 (20.1)	1 (0.3)	138 (45.4)	
**-1.1. Anaemia**								0.228
Carboplatin/PLD	214 (72.8)	25 (8.5)	43 (14.6)	11 (3.7)	1 (0.3)	0 (0.0)	12 (4.1)	
Trabectedin/PLD	232 (76.3)	30 (9.9)	30 (9.9)	11 (3.6)	1 (0.3)	0 (0.0)	12 (3.9)	
**-1.10. Thrombocytopenia**								0.012
Carboplatin/PLD	223 (75.9)	21 (7.1)	18 (6.1)	18 (6.1)	14 (4.8)	0 (0.0)	32 (10.9)	
Trabectedin/PLD	263 (86.5)	11 (3.6)	6 (2.0)	14 (4.6)	9 (3.0)	1 (0.3)	24 (7.9)	
**-1.3. Febrile neutropenia**								0.005
Carboplatin/PLD	291 (99.0)	0 (0.0)	1 (0.3)	0 (0.0)	2 (0.7)	0 (0.0)	2 (0.7)	
Trabectedin/PLD	289 (95.1)	0 (0.0)	0 (0.0)	9 (3.0)	6 (2.0)	0 (0.0)	15 (4.9)	
**-1.4. Granulocytopenia**								0.325
Carboplatin/PLD	294 (100)	0 (0.0)	0 (0.0)	0 (0.0)	0 (0.0)	0 (0.0)	0 (0.0)	
Trabectedin/PLD	303 (99.7)	0 (0.0)	1 (0.3)	0 (0.0)	0 (0.0)	0 (0.0)	0 (0.0)	
**-1.5. Leukopenia**								0.267
Carboplatin/PLD	255 (86.7)	7 (2.4)	17 (5.8)	14 (4.8)	1 (0.3)	0 (0.0)	15 (5.1)	
Trabectedin/PLD	259 (85.2)	8 (2.6)	14 (4.6)	14 (4.6)	8 (2.6)	1 (0.3)	23 (7.6)	
**-1.6. Lymphopenia**								0.026
Carboplatin/PLD	291 (99.0)	3 (1.0)	0 (0.0)	0 (0.0)	0 (0.0)	0 (0.0)	0 (0.0)	
Trabectedin/PLD	296 (97.4)	1 (0.3)	2 (0.7)	3 (1.0)	2 (0.7)	0 (0.0)	5 (1.6)	
**-1.7. Myelosuppression**								0.309
Carboplatin/PLD	293 (99.7)	0 (0.0)	0 (0.0)	1 (0.3)	0 (0.0)	0 (0.0)	1 (0.3)	
Trabectedin/PLD	304 (100)	0 (0.0)	0 (0.0)	0 (0.0)	0 (0.0)	0 (0.0)	0 (0.0)	
**-1.8. Neutropenia**								0.000
Carboplatin/PLD	182 (61.9)	10 (3.4)	35 (11.9)	48 (16.3)	19 (6.5)	0 (0.0)	67 (22.8)	
Trabectedin/PLD	145 (47.7)	14 (4.6)	25 (8.2)	64 (21.1)	56 (18.4)	0 (0.0)	120 (39.5)	
**-1.9. Pancytopenia**								0.246
Carboplatin/PLD	290 (98.6)	1 (0.3)	1 (0.3)	1 (0.3)	1 (0.3)	0 (0.0)	2 (0.7)	
Trabectedin/PLD	303 (99.7)	0 (0.0)	0 (0.0)	1 (0.3)	0 (0.0)	0 (0.0)	1 (0.3)	
**-1.99. Other**								0.064
Carboplatin/PLD	294 (100)	0 (0.0)	0 (0.0)	0 (0.0)	0 (0.0)	0 (0.0)	0 (0.0)	
Trabectedin/PLD	300 (98.7)	1 (0.3)	1 (0.3)	2 (0.7)	0 (0.0)	0 (0.0)	2 (0.7)	
**10. Injury, poisoning and procedural complications**								0.213
Carboplatin/PLD	294 (100)	0 (0.0)	0 (0.0)	0 (0.0)	0 (0.0)	0 (0.0)	0 (0.0)	
Trabectedin/PLD	302 (99.3)	1 (0.3)	0 (0.0)	1 (0.3)	0 (0.0)	0 (0.0)	1 (0.3)	
**-10.99. Other**								0.213
Carboplatin/PLD	294 (100)	0 (0.0)	0 (0.0)	0 (0.0)	0 (0.0)	0 (0.0)	0 (0.0)	

	G0 n (%)	G1 n (%)	G2 n (%)	G3 n (%)	G4 n (%)	G5 n (%)	G3+G4+G5 n (%)	Chi-squared for trend
Trabectedin/PLD	302 (99.3)	1 (0.3)	0 (0.0)	1 (0.3)	0 (0.0)	0 (0.0)	1 (0.3)	
**11. Investigations**								0.117
Carboplatin/PLD	288 (98.0)	3 (1.0)	3 (1.0)	0 (0.0)	0 (0.0)	0 (0.0)	0 (0.0)	
Trabectedin/PLD	291 (95.7)	8 (2.6)	2 (0.7)	2 (0.7)	1 (0.3)	0 (0.0)	3 (1.0)	
**-11.10. Blood lactate dehydrogenase increased**								0.096
Carboplatin/PLD	291 (99.0)	2 (0.7)	1 (0.3)	0 (0.0)	0 (0.0)	0 (0.0)	0 (0.0)	
Trabectedin/PLD	304 (100)	0 (0.0)	0 (0.0)	0 (0.0)	0 (0.0)	0 (0.0)	0 (0.0)	
**-11.25. Weight decreased**								0.500
Carboplatin/PLD	293 (99.7)	0 (0.0)	1 (0.3)	0 (0.0)	0 (0.0)	0 (0.0)	0 (0.0)	
Trabectedin/PLD	300 (98.7)	4 (1.3)	0 (0.0)	0 (0.0)	0 (0.0)	0 (0.0)	0 (0.0)	
**-11.30 Blood bilirubin decreased**								0.309
Carboplatin/PLD	293 (99.7)	1 (0.3)	0 (0.0)	0 (0.0)	0 (0.0)	0 (0.0)	0 (0.0)	
Trabectedin/PLD	304 (100)	0 (0.0)	0 (0.0)	0 (0.0)	0 (0.0)	0 (0.0)	0 (0.0)	
**-11.4. Blood albumin decreased**								0.325
Carboplatin/PLD	294 (100)	0 (0.0)	0 (0.0)	0 (0.0)	0 (0.0)	0 (0.0)	0 (0.0)	
Trabectedin/PLD	303 (99.7)	0 (0.0)	1 (0.3)	0 (0.0)	0 (0.0)	0 (0.0)	0 (0.0)	
**-11.7. Blood creatine phosphokinase increased**								0.109
Carboplatin/PLD	293 (99.7)	1 (0.3)	0 (0.0)	0 (0.0)	0 (0.0)	0 (0.0)	0 (0.0)	
Trabectedin/PLD	299 (98.4)	3 (1.0)	1 (0.3)	0 (0.0)	1 (0.3)	0 (0.0)	1 (0.3)	
**-11.8. Blood creatinine decreased**								0.325
Carboplatin/PLD	294 (100)	0 (0.0)	0 (0.0)	0 (0.0)	0 (0.0)	0 (0.0)	0 (0.0)	
Trabectedin/PLD	303 (99.7)	1 (0.3)	0 (0.0)	0 (0.0)	0 (0.0)	0 (0.0)	0 (0.0)	
**-11.99. Other**								0.409
Carboplatin/PLD	293 (99.7)	0 (0.0)	1 (0.3)	0 (0.0)	0 (0.0)	0 (0.0)	0 (0.0)	
Trabectedin/PLD	302 (99.3)	0 (0.0)	0 (0.0)	2 (0.7)	0 (0.0)	0 (0.0)	2 (0.7)	
**12. Metabolism and nutrition disorders**								0.085
Carboplatin/PLD	267 (90.8)	17 (5.8)	7 (2.4)	3 (1.0)	0 (0.0)	0 (0.0)	3 (1.0)	
Trabectedin/PLD	267 (87.8)	18 (5.9)	9 (3.0)	10 (3.3)	0 (0.0)	0 (0.0)	10 (3.3)	
**-12.10. Hypocalcaemia**								0.583
Carboplatin/PLD	293 (99.7)	1 (0.3)	0 (0.0)	0 (0.0)	0 (0.0)	0 (0.0)	0 (0.0)	
Trabectedin/PLD	302 (99.3)	2 (0.7)	0 (0.0)	0 (0.0)	0 (0.0)	0 (0.0)	0 (0.0)	
**-12.11. Hypokalaemia**								0.332
Carboplatin/PLD	293 (99.7)	0 (0.0)	0 (0.0)	1 (0.3)	0 (0.0)	0 (0.0)	1 (0.3)	
Trabectedin/PLD	301 (99.0)	0 (0.0)	0 (0.0)	3 (1.0)	0 (0.0)	0 (0.0)	3 (1.0)	
**-12.12 Hypomagnesaemia**								0.209
Carboplatin/PLD	292 (99.3)	2 (0.7)	0 (0.0)	0 (0.0)	0 (0.0)	0 (0.0)	0 (0.0)	
Trabectedin/PLD	299 (98.4)	4 (1.3)	0 (0.0)	1 (0.3)	0 (0.0)	0 (0.0)	1 (0.3)	
**-12.13 Hyponatraemia**								0.423
Carboplatin/PLD	292 (99.3)	2 (0.7)	0 (0.0)	0 (0.0)	0 (0.0)	0 (0.0)	0 (0.0)	
Trabectedin/PLD	301 (99.0)	2 (0.7)	0 (0.0)	1 (0.3)	0 (0.0)	0 (0.0)	1 (0.3)	
**-12.15. Hypophosphatemia**								0.583
Carboplatin/PLD	292 (99.3)	2 (0.7)	0 (0.0)	0 (0.0)	0 (0.0)	0 (0.0)	0 (0.0)	
Trabectedin/PLD	302 (99.3)	1 (0.3)	0 (0.0)	1 (0.3)	0 (0.0)	0 (0.0)	1 (0.3)	
**-12.2. Appetite disorder**								0.294

	G0 n (%)	G1 n (%)	G2 n (%)	G3 n (%)	G4 n (%)	G5 n (%)	G3+G4+G5 n (%)	Chi-squared for trend
Carboplatin/PLD	271 (92.2)	14 (4.8)	7 (2.4)	2 (0.7)	0 (0.0)	0 (0.0)	2 (0.7)	
Trabectedin/PLD	275 (90.5)	15 (4.9)	9 (3.0)	5 (1.6)	0 (0.0)	0 (0.0)	5 (1.6)	
**-12.5. Hypercalcaemia**								0.325
Carboplatin/PLD	294 (100)	0 (0.0)	0 (0.0)	0 (0.0)	0 (0.0)	0 (0.0)	0 (0.0)	
Trabectedin/PLD	303 (99.7)	0 (0.0)	1 (0.3)	0 (0.0)	0 (0.0)	0 (0.0)	0 (0.0)	
**-12.6. Hyperglycaemia**								0.973
Carboplatin/PLD	292 (99.3)	2 (0.7)	0 (0.0)	0 (0.0)	0 (0.0)	0 (0.0)	0 (0.0)	
Trabectedin/PLD	302 (99.3)	2 (0.7)	0 (0.0)	0 (0.0)	0 (0.0)	0 (0.0)	0 (0.0)	
**-12.7. Hypermagnesaemia**								0.325
Carboplatin/PLD	294 (100)	0 (0.0)	0 (0.0)	0 (0.0)	0 (0.0)	0 (0.0)	0 (0.0)	
Trabectedin/PLD	303 (99.7)	1 (0.3)	0 (0.0)	0 (0.0)	0 (0.0)	0 (0.0)	0 (0.0)	
**-12.8. Hypernatraemia**								0.309
Carboplatin/PLD	293 (99.7)	1 (0.3)	0 (0.0)	0 (0.0)	0 (0.0)	0 (0.0)	0 (0.0)	
Trabectedin/PLD	304 (100)	0 (0.0)	0 (0.0)	0 (0.0)	0 (0.0)	0 (0.0)	0 (0.0)	
**-12.99. Other**								0.150
Carboplatin/PLD	292 (99.3)	2 (0.7)	0 (0.0)	0 (0.0)	0 (0.0)	0 (0.0)	0 (0.0)	
Trabectedin/PLD	304 (100)	0 (0.0)	0 (0.0)	0 (0.0)	0 (0.0)	0 (0.0)	0 (0.0)	
**13. Musculoskeletal and connective tissue disorders**								0.285
Carboplatin/PLD	281 (95.6)	9 (3.1)	4 (1.4)	0 (0.0)	0 (0.0)	0 (0.0)	0 (0.0)	
Trabectedin/PLD	284 (93.4)	14 (4.6)	6 (2.0)	0 (0.0)	0 (0.0)	0 (0.0)	0 (0.0)	
**-13.1. Arthralgia**								0.050
Carboplatin/PLD	290 (98.6)	2 (0.7)	2 (0.7)	0 (0.0)	0 (0.0)	0 (0.0)	0 (0.0)	
Trabectedin/PLD	290 (95.4)	10 (3.3)	4 (1.3)	0 (0.0)	0 (0.0)	0 (0.0)	0 (0.0)	
**-13.12. Myalgia**								0.950
Carboplatin/PLD	287 (97.6)	5 (1.7)	2 (0.7)	0 (0.0)	0 (0.0)	0 (0.0)	0 (0.0)	
Trabectedin/PLD	296 (97.4)	7 (2.3)	1 (0.3)	0 (0.0)	0 (0.0)	0 (0.0)	0 (0.0)	
**-13.3. Bone pain**								0.309
Carboplatin/PLD	293 (99.7)	1 (0.3)	0 (0.0)	0 (0.0)	0 (0.0)	0 (0.0)	0 (0.0)	
Trabectedin/PLD	304 (100)	0 (0.0)	0 (0.0)	0 (0.0)	0 (0.0)	0 (0.0)	0 (0.0)	
**-13.8. Muscular weakness**								0.325
Carboplatin/PLD	294 (100)	0 (0.0)	0 (0.0)	0 (0.0)	0 (0.0)	0 (0.0)	0 (0.0)	
Trabectedin/PLD	303 (99.7)	0 (0.0)	1 (0.3)	0 (0.0)	0 (0.0)	0 (0.0)	0 (0.0)	
**-13.99. Other**								0.041
Carboplatin/PLD	290 (98.6)	4 (1.4)	0 (0.0)	0 (0.0)	0 (0.0)	0 (0.0)	0 (0.0)	
Trabectedin/PLD	304 (100)	0 (0.0)	0 (0.0)	0 (0.0)	0 (0.0)	0 (0.0)	0 (0.0)	
**14. Neoplasms benign, malignant and unspecified (incl cysts and polyps)**								0.186
Carboplatin/PLD	292 (99.3)	0 (0.0)	1 (0.3)	0 (0.0)	0 (0.0)	1 (0.3)	1 (0.3)	
Trabectedin/PLD	304 (100)	0 (0.0)	0 (0.0)	0 (0.0)	0 (0.0)	0 (0.0)	0 (0.0)	
**-14.2. Cancer pain**								0.309
Carboplatin/PLD	293 (99.7)	0 (0.0)	1 (0.3)	0 (0.0)	0 (0.0)	0 (0.0)	0 (0.0)	
Trabectedin/PLD	304 (100)	0 (0.0)	0 (0.0)	0 (0.0)	0 (0.0)	0 (0.0)	0 (0.0)	
**-14.99. Other**								0.309
Carboplatin/PLD	293 (99.7)	0 (0.0)	0 (0.0)	0 (0.0)	0 (0.0)	1 (0.3)	1 (0.3)	

	G0<br>n (%)	G1<br>n (%)	G2<br>n (%)	G3<br>n (%)	G4<br>n (%)	G5<br>n (%)	G3+G4+G5<br>n (%)	Chi-squared for trend
Trabectedin/PLD	304 (100)	0 (0.0)	0 (0.0)	0 (0.0)	0 (0.0)	0 (0.0)	0 (0.0)	
**15. Nervous system disorders**								0.868
Carboplatin/PLD	246 (83.7)	30 (10.2)	17 (5.8)	1 (0.3)	0 (0.0)	0 (0.0)	1 (0.3)	
Trabectedin/PLD	254 (83.6)	35 (11.5)	13 (4.3)	2 (0.7)	0 (0.0)	0 (0.0)	2 (0.7)	
**-15.1. Dizziness**								0.942
Carboplatin/PLD	283 (96.3)	6 (2.0)	5 (1.7)	0 (0.0)	0 (0.0)	0 (0.0)	0 (0.0)	
Trabectedin/PLD	294 (96.7)	5 (1.6)	4 (1.3)	1 (0.3)	0 (0.0)	0 (0.0)	1 (0.3)	
**-15.11. Presyncope**								0.325
Carboplatin/PLD	294 (100)	0 (0.0)	0 (0.0)	0 (0.0)	0 (0.0)	0 (0.0)	0 (0.0)	
Trabectedin/PLD	303 (99.7)	0 (0.0)	1 (0.3)	0 (0.0)	0 (0.0)	0 (0.0)	0 (0.0)	
**-15.12. Syncope**								0.309
Carboplatin/PLD	293 (99.7)	0 (0.0)	1 (0.3)	0 (0.0)	0 (0.0)	0 (0.0)	0 (0.0)	
Trabectedin/PLD	304 (100)	0 (0.0)	0 (0.0)	0 (0.0)	0 (0.0)	0 (0.0)	0 (0.0)	
**-15.2. Dysgeusia**								0.810
Carboplatin/PLD	285 (96.9)	5 (1.7)	4 (1.4)	0 (0.0)	0 (0.0)	0 (0.0)	0 (0.0)	
Trabectedin/PLD	292 (96.1)	9 (3.0)	3 (1.0)	0 (0.0)	0 (0.0)	0 (0.0)	0 (0.0)	
**-15.3. Headache**								0.115
Carboplatin/PLD	286 (97.3)	8 (2.7)	0 (0.0)	0 (0.0)	0 (0.0)	0 (0.0)	0 (0.0)	
Trabectedin/PLD	301 (99.0)	3 (1.0)	0 (0.0)	0 (0.0)	0 (0.0)	0 (0.0)	0 (0.0)	
**-15.5. Insomnia**								0.416
Carboplatin/PLD	291 (99.0)	2 (0.7)	1 (0.3)	0 (0.0)	0 (0.0)	0 (0.0)	0 (0.0)	
Trabectedin/PLD	299 (98.4)	3 (1.0)	1 (0.3)	1 (0.3)	0 (0.0)	0 (0.0)	1 (0.3)	
**-15.6. Memory impairment**								0.309
Carboplatin/PLD	293 (99.7)	1 (0.3)	0 (0.0)	0 (0.0)	0 (0.0)	0 (0.0)	0 (0.0)	
Trabectedin/PLD	304 (100)	0 (0.0)	0 (0.0)	0 (0.0)	0 (0.0)	0 (0.0)	0 (0.0)	
**-15.7. Neuropathy**								0.345
Carboplatin/PLD	268 (91.2)	16 (5.4)	9 (3.1)	1 (0.3)	0 (0.0)	0 (0.0)	1 (0.3)	
Trabectedin/PLD	280 (92.1)	19 (6.3)	5 (1.6)	0 (0.0)	0 (0.0)	0 (0.0)	0 (0.0)	
**-15.99. Other**								0.211
Carboplatin/PLD	292 (99.3)	1 (0.3)	1 (0.3)	0 (0.0)	0 (0.0)	0 (0.0)	0 (0.0)	
Trabectedin/PLD	297 (97.7)	6 (2.0)	1 (0.3)	0 (0.0)	0 (0.0)	0 (0.0)	0 (0.0)	
**16. Psychiatric disorders**								0.379
Carboplatin/PLD	293 (99.7)	1 (0.3)	0 (0.0)	0 (0.0)	0 (0.0)	0 (0.0)	0 (0.0)	
Trabectedin/PLD	302 (99.3)	1 (0.3)	0 (0.0)	1 (0.3)	0 (0.0)	0 (0.0)	1 (0.3)	
**-16.2. Altered state of consciousness**								0.309
Carboplatin/PLD	293 (99.7)	1 (0.3)	0 (0.0)	0 (0.0)	0 (0.0)	0 (0.0)	0 (0.0)	
Trabectedin/PLD	304 (100)	0 (0.0)	0 (0.0)	0 (0.0)	0 (0.0)	0 (0.0)	0 (0.0)	
**-16.3. Anxiety**								0.325
Carboplatin/PLD	294 (100)	0 (0.0)	0 (0.0)	0 (0.0)	0 (0.0)	0 (0.0)	0 (0.0)	
Trabectedin/PLD	303 (99.7)	1 (0.3)	0 (0.0)	0 (0.0)	0 (0.0)	0 (0.0)	0 (0.0)	
**-16.4. Depressed mood**								0.325
Carboplatin/PLD	294 (100)	0 (0.0)	0 (0.0)	0 (0.0)	0 (0.0)	0 (0.0)	0 (0.0)	
Trabectedin/PLD	303 (99.7)	0 (0.0)	0 (0.0)	1 (0.3)	0 (0.0)	0 (0.0)	1 (0.3)	
**17. Renal and urinary disorders**								0.521

	G0 n (%)	G1 n (%)	G2 n (%)	G3 n (%)	G4 n (%)	G5 n (%)	G3+G4+G5 n (%)	Chi-squared for trend
Carboplatin/PLD	285 (96.9)	5 (1.7)	3 (1.0)	1 (0.3)	0 (0.0)	0 (0.0)	1 (0.3)	
Trabectedin/PLD	297 (97.7)	6 (2.0)	0 (0.0)	0 (0.0)	1 (0.3)	0 (0.0)	1 (0.3)	
**-17.2. Dysuria**								0.164
Carboplatin/PLD	294 (100)	0 (0.0)	0 (0.0)	0 (0.0)	0 (0.0)	0 (0.0)	0 (0.0)	
Trabectedin/PLD	302 (99.3)	2 (0.7)	0 (0.0)	0 (0.0)	0 (0.0)	0 (0.0)	0 (0.0)	
**-17.3. Flank pain**								0.638
Carboplatin/PLD	293 (99.7)	0 (0.0)	1 (0.3)	0 (0.0)	0 (0.0)	0 (0.0)	0 (0.0)	
Trabectedin/PLD	303 (99.7)	1 (0.3)	0 (0.0)	0 (0.0)	0 (0.0)	0 (0.0)	0 (0.0)	
**-17.5. Renal failure**								0.814
Carboplatin/PLD	288 (98.0)	5 (1.7)	1 (0.3)	0 (0.0)	0 (0.0)	0 (0.0)	0 (0.0)	
Trabectedin/PLD	301 (99.0)	2 (0.7)	0 (0.0)	0 (0.0)	1 (0.3)	0 (0.0)	1 (0.3)	
**-17.8. Urinary incontinence**								0.325
Carboplatin/PLD	294 (100)	0 (0.0)	0 (0.0)	0 (0.0)	0 (0.0)	0 (0.0)	0 (0.0)	
Trabectedin/PLD	303 (99.7)	1 (0.3)	0 (0.0)	0 (0.0)	0 (0.0)	0 (0.0)	0 (0.0)	
**-17.99. Other**								0.593
Carboplatin/PLD	292 (99.3)	0 (0.0)	1 (0.3)	1 (0.3)	0 (0.0)	0 (0.0)	1 (0.3)	
Trabectedin/PLD	301 (99.0)	3 (1.0)	0 (0.0)	0 (0.0)	0 (0.0)	0 (0.0)	0 (0.0)	
**18. Reproductive system and breast disorders**								0.799
Carboplatin/PLD	293 (99.7)	0 (0.0)	1 (0.3)	0 (0.0)	0 (0.0)	0 (0.0)	0 (0.0)	
Trabectedin/PLD	303 (99.7)	0 (0.0)	0 (0.0)	1 (0.3)	0 (0.0)	0 (0.0)	1 (0.3)	
**-18.2. Genital herpes**								0.309
Carboplatin/PLD	293 (99.7)	0 (0.0)	1 (0.3)	0 (0.0)	0 (0.0)	0 (0.0)	0 (0.0)	
Trabectedin/PLD	304 (100)	0 (0.0)	0 (0.0)	0 (0.0)	0 (0.0)	0 (0.0)	0 (0.0)	
**-18.5. Vulvar erosion**								0.309
Carboplatin/PLD	293 (99.7)	0 (0.0)	1 (0.3)	0 (0.0)	0 (0.0)	0 (0.0)	0 (0.0)	
Trabectedin/PLD	304 (100)	0 (0.0)	0 (0.0)	0 (0.0)	0 (0.0)	0 (0.0)	0 (0.0)	
**-18.99. Other**								0.325
Carboplatin/PLD	294 (100)	0 (0.0)	0 (0.0)	0 (0.0)	0 (0.0)	0 (0.0)	0 (0.0)	
Trabectedin/PLD	303 (99.7)	0 (0.0)	0 (0.0)	1 (0.3)	0 (0.0)	0 (0.0)	1 (0.3)	
**19. Respiratory, thoracic and mediastinal disorders**								0.001
Carboplatin/PLD	277 (94.2)	13 (4.4)	3 (1.0)	1 (0.3)	0 (0.0)	0 (0.0)	1 (0.3)	
Trabectedin/PLD	268 (88.2)	16 (5.3)	10 (3.3)	7 (2.3)	2 (0.7)	1 (0.3)	10 (3.3)	
**-19.12. Pharyngolaryngeal pain**								0.332
Carboplatin/PLD	293 (99.7)	1 (0.3)	0 (0.0)	0 (0.0)	0 (0.0)	0 (0.0)	0 (0.0)	
Trabectedin/PLD	301 (99.0)	3 (1.0)	0 (0.0)	0 (0.0)	0 (0.0)	0 (0.0)	0 (0.0)	
**-19.13. Pulmonary embolism**								0.091
Carboplatin/PLD	294 (100)	0 (0.0)	0 (0.0)	0 (0.0)	0 (0.0)	0 (0.0)	0 (0.0)	
Trabectedin/PLD	301 (99.0)	0 (0.0)	0 (0.0)	2 (0.7)	1 (0.3)	0 (0.0)	3 (1.0)	
**-19.16. Respiratory tract infection**								0.325
Carboplatin/PLD	294 (100)	0 (0.0)	0 (0.0)	0 (0.0)	0 (0.0)	0 (0.0)	0 (0.0)	
Trabectedin/PLD	303 (99.7)	0 (0.0)	1 (0.3)	0 (0.0)	0 (0.0)	0 (0.0)	0 (0.0)	
**-19.17. Rhinitis allergic**								0.429
Carboplatin/PLD	293 (99.7)	1 (0.3)	0 (0.0)	0 (0.0)	0 (0.0)	0 (0.0)	0 (0.0)	

	G0 n (%)	G1 n (%)	G2 n (%)	G3 n (%)	G4 n (%)	G5 n (%)	G3+G4+G5 n (%)	Chi-squared for trend
Trabectedin/PLD	302 (99.3)	1 (0.3)	1 (0.3)	0 (0.0)	0 (0.0)	0 (0.0)	0 (0.0)	
**-19.2. Bronchitis**								0.325
Carboplatin/PLD	294 (100)	0 (0.0)	0 (0.0)	0 (0.0)	0 (0.0)	0 (0.0)	0 (0.0)	
Trabectedin/PLD	303 (99.7)	0 (0.0)	0 (0.0)	1 (0.3)	0 (0.0)	0 (0.0)	1 (0.3)	
**-19.3. Cough**								0.303
Carboplatin/PLD	292 (99.3)	1 (0.3)	1 (0.3)	0 (0.0)	0 (0.0)	0 (0.0)	0 (0.0)	
Trabectedin/PLD	298 (98.0)	5 (1.6)	1 (0.3)	0 (0.0)	0 (0.0)	0 (0.0)	0 (0.0)	
**-19.5. Dyspnoea**								0.002
Carboplatin/PLD	289 (98.3)	3 (1.0)	2 (0.7)	0 (0.0)	0 (0.0)	0 (0.0)	0 (0.0)	
Trabectedin/PLD	282 (92.8)	12 (3.9)	6 (2.0)	3 (1.0)	1 (0.3)	0 (0.0)	4 (1.3)	
**-19.6. Epistaxis**								0.095
Carboplatin/PLD	286 (97.3)	7 (2.4)	0 (0.0)	1 (0.3)	0 (0.0)	0 (0.0)	1 (0.3)	
Trabectedin/PLD	301 (99.0)	3 (1.0)	0 (0.0)	0 (0.0)	0 (0.0)	0 (0.0)	0 (0.0)	
**-19.9. Nasopharyngitis**								0.309
Carboplatin/PLD	293 (99.7)	1 (0.3)	0 (0.0)	0 (0.0)	0 (0.0)	0 (0.0)	0 (0.0)	
Trabectedin/PLD	304 (100)	0 (0.0)	0 (0.0)	0 (0.0)	0 (0.0)	0 (0.0)	0 (0.0)	
**-19.99. Other**								0.141
Carboplatin/PLD	291 (99.0)	3 (1.0)	0 (0.0)	0 (0.0)	0 (0.0)	0 (0.0)	0 (0.0)	
Trabectedin/PLD	298 (98.0)	3 (1.0)	1 (0.3)	1 (0.3)	0 (0.0)	1 (0.3)	2 (0.7)	
**2. Cardiac disorders**								0.023
Carboplatin/PLD	293 (99.7)	1 (0.3)	0 (0.0)	0 (0.0)	0 (0.0)	0 (0.0)	0 (0.0)	
Trabectedin/PLD	297 (97.7)	1 (0.3)	3 (1.0)	2 (0.7)	1 (0.3)	0 (0.0)	3 (1.0)	
**-2.2. Left ventricular dysfunction**								0.325
Carboplatin/PLD	294 (100)	0 (0.0)	0 (0.0)	0 (0.0)	0 (0.0)	0 (0.0)	0 (0.0)	
Trabectedin/PLD	303 (99.7)	0 (0.0)	0 (0.0)	1 (0.3)	0 (0.0)	0 (0.0)	1 (0.3)	
**-2.3. Palpitations**								0.216
Carboplatin/PLD	293 (99.7)	1 (0.3)	0 (0.0)	0 (0.0)	0 (0.0)	0 (0.0)	0 (0.0)	
Trabectedin/PLD	301 (99.0)	1 (0.3)	2 (0.7)	0 (0.0)	0 (0.0)	0 (0.0)	0 (0.0)	
**-2.4. Tachycardia**								0.172
Carboplatin/PLD	294 (100)	0 (0.0)	0 (0.0)	0 (0.0)	0 (0.0)	0 (0.0)	0 (0.0)	
Trabectedin/PLD	302 (99.3)	0 (0.0)	1 (0.3)	1 (0.3)	0 (0.0)	0 (0.0)	1 (0.3)	
**-2.99. Other**								0.187
Carboplatin/PLD	294 (100)	0 (0.0)	0 (0.0)	0 (0.0)	0 (0.0)	0 (0.0)	0 (0.0)	
Trabectedin/PLD	302 (99.3)	0 (0.0)	1 (0.3)	0 (0.0)	1 (0.3)	0 (0.0)	1 (0.3)	
**20. Skin and subcutaneous tissue disorders**								0.290
Carboplatin/PLD	230 (78.2)	43 (14.6)	17 (5.8)	4 (1.4)	0 (0.0)	0 (0.0)	4 (1.4)	
Trabectedin/PLD	229 (75.3)	49 (16.1)	17 (5.6)	9 (3.0)	0 (0.0)	0 (0.0)	9 (3.0)	
**-20.1. Alopecia**								0.775
Carboplatin/PLD	282 (95.9)	11 (3.7)	1 (0.3)	0 (0.0)	0 (0.0)	0 (0.0)	0 (0.0)	
Trabectedin/PLD	289 (95.1)	15 (4.9)	0 (0.0)	0 (0.0)	0 (0.0)	0 (0.0)	0 (0.0)	
**-20.10. Hyperhidrosis**								0.423
Carboplatin/PLD	293 (99.7)	0 (0.0)	1 (0.3)	0 (0.0)	0 (0.0)	0 (0.0)	0 (0.0)	
Trabectedin/PLD	301 (99.0)	1 (0.3)	2 (0.7)	0 (0.0)	0 (0.0)	0 (0.0)	0 (0.0)	
**-20.12. Nail disorder**								0.102

	G0 n (%)	G1 n (%)	G2 n (%)	G3 n (%)	G4 n (%)	G5 n (%)	G3+G4+G5 n (%)	Chi-squared for trend
Carboplatin/PLD	293 (99.7)	1 (0.3)	0 (0.0)	0 (0.0)	0 (0.0)	0 (0.0)	0 (0.0)	
Trabectedin/PLD	299 (98.4)	4 (1.3)	1 (0.3)	0 (0.0)	0 (0.0)	0 (0.0)	0 (0.0)	
**-20.13. Night sweats**								0.141
Carboplatin/PLD	293 (99.7)	1 (0.3)	0 (0.0)	0 (0.0)	0 (0.0)	0 (0.0)	0 (0.0)	
Trabectedin/PLD	300 (98.7)	2 (0.7)	1 (0.3)	1 (0.3)	0 (0.0)	0 (0.0)	1 (0.3)	
**-20.14. Palmar-plantar erythrodysaesthesia syndrome**								0.111
Carboplatin/PLD	272 (92.5)	12 (4.1)	9 (3.1)	1 (0.3)	0 (0.0)	0 (0.0)	1 (0.3)	
Trabectedin/PLD	271 (89.1)	17 (5.6)	11 (3.6)	5 (1.6)	0 (0.0)	0 (0.0)	5 (1.6)	
**-20.15. Pigmentation disorder**								0.602
Carboplatin/PLD	289 (98.3)	4 (1.4)	0 (0.0)	1 (0.3)	0 (0.0)	0 (0.0)	1 (0.3)	
Trabectedin/PLD	299 (98.4)	5 (1.6)	0 (0.0)	0 (0.0)	0 (0.0)	0 (0.0)	0 (0.0)	
**-20.16. Pruritus**								0.290
Carboplatin/PLD	287 (97.6)	6 (2.0)	1 (0.3)	0 (0.0)	0 (0.0)	0 (0.0)	0 (0.0)	
Trabectedin/PLD	301 (99.0)	2 (0.7)	1 (0.3)	0 (0.0)	0 (0.0)	0 (0.0)	0 (0.0)	
**-20.17. Purpura**								0.309
Carboplatin/PLD	293 (99.7)	1 (0.3)	0 (0.0)	0 (0.0)	0 (0.0)	0 (0.0)	0 (0.0)	
Trabectedin/PLD	304 (100)	0 (0.0)	0 (0.0)	0 (0.0)	0 (0.0)	0 (0.0)	0 (0.0)	
**-20.18. Rash**								0.617
Carboplatin/PLD	287 (97.6)	4 (1.4)	2 (0.7)	1 (0.3)	0 (0.0)	0 (0.0)	1 (0.3)	
Trabectedin/PLD	295 (97.0)	5 (1.6)	2 (0.7)	2 (0.7)	0 (0.0)	0 (0.0)	2 (0.7)	
**-20.2. Catheter site erythema**								0.164
Carboplatin/PLD	294 (100)	0 (0.0)	0 (0.0)	0 (0.0)	0 (0.0)	0 (0.0)	0 (0.0)	
Trabectedin/PLD	302 (99.3)	2 (0.7)	0 (0.0)	0 (0.0)	0 (0.0)	0 (0.0)	0 (0.0)	
**-20.20. Skin irritation**								0.309
Carboplatin/PLD	293 (99.7)	1 (0.3)	0 (0.0)	0 (0.0)	0 (0.0)	0 (0.0)	0 (0.0)	
Trabectedin/PLD	304 (100)	0 (0.0)	0 (0.0)	0 (0.0)	0 (0.0)	0 (0.0)	0 (0.0)	
**-20.21. Skin toxicity**								0.041
Carboplatin/PLD	290 (98.6)	4 (1.4)	0 (0.0)	0 (0.0)	0 (0.0)	0 (0.0)	0 (0.0)	
Trabectedin/PLD	304 (100)	0 (0.0)	0 (0.0)	0 (0.0)	0 (0.0)	0 (0.0)	0 (0.0)	
**-20.22 Urticaria**								0.309
Carboplatin/PLD	293 (99.7)	1 (0.3)	0 (0.0)	0 (0.0)	0 (0.0)	0 (0.0)	0 (0.0)	
Trabectedin/PLD	304 (100)	0 (0.0)	0 (0.0)	0 (0.0)	0 (0.0)	0 (0.0)	0 (0.0)	
**-20.5. Dermatitis**								0.735
Carboplatin/PLD	291 (99.0)	2 (0.7)	1 (0.3)	0 (0.0)	0 (0.0)	0 (0.0)	0 (0.0)	
Trabectedin/PLD	302 (99.3)	1 (0.3)	1 (0.3)	0 (0.0)	0 (0.0)	0 (0.0)	0 (0.0)	
**-20.6. Dermatitis acneiform**								0.981
Carboplatin/PLD	293 (99.7)	1 (0.3)	0 (0.0)	0 (0.0)	0 (0.0)	0 (0.0)	0 (0.0)	
Trabectedin/PLD	303 (99.7)	1 (0.3)	0 (0.0)	0 (0.0)	0 (0.0)	0 (0.0)	0 (0.0)	
**-20.7. Dry skin**								0.213
Carboplatin/PLD	281 (95.6)	10 (3.4)	2 (0.7)	1 (0.3)	0 (0.0)	0 (0.0)	1 (0.3)	
Trabectedin/PLD	294 (96.7)	10 (3.3)	0 (0.0)	0 (0.0)	0 (0.0)	0 (0.0)	0 (0.0)	
**-20.8. Erythema**								0.686
Carboplatin/PLD	290 (98.6)	3 (1.0)	1 (0.3)	0 (0.0)	0 (0.0)	0 (0.0)	0 (0.0)	

	G0 n (%)	G1 n (%)	G2 n (%)	G3 n (%)	G4 n (%)	G5 n (%)	G3+G4+G5 n (%)	Chi-squared for trend
Trabectedin/PLD	299 (98.4)	4 (1.3)	0 (0.0)	1 (0.3)	0 (0.0)	0 (0.0)	1 (0.3)	
**-20.99. Other**								0.035
Carboplatin/PLD	293 (99.7)	1 (0.3)	0 (0.0)	0 (0.0)	0 (0.0)	0 (0.0)	0 (0.0)	
Trabectedin/PLD	297 (97.7)	5 (1.6)	2 (0.7)	0 (0.0)	0 (0.0)	0 (0.0)	0 (0.0)	
**21. Vascular disorders**								0.002
Carboplatin/PLD	287 (97.6)	5 (1.7)	2 (0.7)	0 (0.0)	0 (0.0)	0 (0.0)	0 (0.0)	
Trabectedin/PLD	282 (92.8)	9 (3.0)	11 (3.6)	2 (0.7)	0 (0.0)	0 (0.0)	2 (0.7)	
**-21.2. Flushing**								0.222
Carboplatin/PLD	291 (99.0)	3 (1.0)	0 (0.0)	0 (0.0)	0 (0.0)	0 (0.0)	0 (0.0)	
Trabectedin/PLD	297 (97.7)	7 (2.3)	0 (0.0)	0 (0.0)	0 (0.0)	0 (0.0)	0 (0.0)	
**-21.4. Hypertension**								0.631
Carboplatin/PLD	290 (98.6)	2 (0.7)	2 (0.7)	0 (0.0)	0 (0.0)	0 (0.0)	0 (0.0)	
Trabectedin/PLD	300 (98.7)	1 (0.3)	1 (0.3)	2 (0.7)	0 (0.0)	0 (0.0)	2 (0.7)	
**-21.5. Hypotension**								0.108
Carboplatin/PLD	294 (100)	0 (0.0)	0 (0.0)	0 (0.0)	0 (0.0)	0 (0.0)	0 (0.0)	
Trabectedin/PLD	301 (99.0)	2 (0.7)	1 (0.3)	0 (0.0)	0 (0.0)	0 (0.0)	0 (0.0)	
**-21.6. Lymphoedema**								0.309
Carboplatin/PLD	293 (99.7)	1 (0.3)	0 (0.0)	0 (0.0)	0 (0.0)	0 (0.0)	0 (0.0)	
Trabectedin/PLD	304 (100)	0 (0.0)	0 (0.0)	0 (0.0)	0 (0.0)	0 (0.0)	0 (0.0)	
**-21.8. Phlebitis**								0.049
Carboplatin/PLD	294 (100)	0 (0.0)	0 (0.0)	0 (0.0)	0 (0.0)	0 (0.0)	0 (0.0)	
Trabectedin/PLD	300 (98.7)	0 (0.0)	4 (1.3)	0 (0.0)	0 (0.0)	0 (0.0)	0 (0.0)	
**-21.9. Thrombosis**								0.031
Carboplatin/PLD	294 (100)	0 (0.0)	0 (0.0)	0 (0.0)	0 (0.0)	0 (0.0)	0 (0.0)	
Trabectedin/PLD	299 (98.4)	1 (0.3)	4 (1.3)	0 (0.0)	0 (0.0)	0 (0.0)	0 (0.0)	
**-21.99. Other**								0.671
Carboplatin/PLD	293 (99.7)	1 (0.3)	0 (0.0)	0 (0.0)	0 (0.0)	0 (0.0)	0 (0.0)	
Trabectedin/PLD	303 (99.7)	0 (0.0)	1 (0.3)	0 (0.0)	0 (0.0)	0 (0.0)	0 (0.0)	
**22. Other**								0.172
Carboplatin/PLD	292 (99.3)	1 (0.3)	1 (0.3)	0 (0.0)	0 (0.0)	0 (0.0)	0 (0.0)	
Trabectedin/PLD	304 (100)	0 (0.0)	0 (0.0)	0 (0.0)	0 (0.0)	0 (0.0)	0 (0.0)	
**3. Ear and labyrinth disorders**								0.970
Carboplatin/PLD	290 (98.6)	3 (1.0)	1 (0.3)	0 (0.0)	0 (0.0)	0 (0.0)	0 (0.0)	
Trabectedin/PLD	302 (99.3)	0 (0.0)	1 (0.3)	1 (0.3)	0 (0.0)	0 (0.0)	1 (0.3)	
**-3.1. Ear discomfort**								0.309
Carboplatin/PLD	293 (99.7)	1 (0.3)	0 (0.0)	0 (0.0)	0 (0.0)	0 (0.0)	0 (0.0)	
Trabectedin/PLD	304 (100)	0 (0.0)	0 (0.0)	0 (0.0)	0 (0.0)	0 (0.0)	0 (0.0)	
**-3.2. Hypoacusis**								0.325
Carboplatin/PLD	294 (100)	0 (0.0)	0 (0.0)	0 (0.0)	0 (0.0)	0 (0.0)	0 (0.0)	
Trabectedin/PLD	303 (99.7)	0 (0.0)	1 (0.3)	0 (0.0)	0 (0.0)	0 (0.0)	0 (0.0)	
**-3.3. Ototoxicity**								0.309
Carboplatin/PLD	293 (99.7)	0 (0.0)	1 (0.3)	0 (0.0)	0 (0.0)	0 (0.0)	0 (0.0)	
Trabectedin/PLD	304 (100)	0 (0.0)	0 (0.0)	0 (0.0)	0 (0.0)	0 (0.0)	0 (0.0)	
**-3.4. Tinnitus**								0.150

	G0 n (%)	G1 n (%)	G2 n (%)	G3 n (%)	G4 n (%)	G5 n (%)	G3+G4+G5 n (%)	Chi-squared for trend
Carboplatin/PLD	292 (99.3)	2 (0.7)	0 (0.0)	0 (0.0)	0 (0.0)	0 (0.0)	0 (0.0)	
Trabectedin/PLD	304 (100)	0 (0.0)	0 (0.0)	0 (0.0)	0 (0.0)	0 (0.0)	0 (0.0)	
**-3.99. Other**								0.325
Carboplatin/PLD	294 (100)	0 (0.0)	0 (0.0)	0 (0.0)	0 (0.0)	0 (0.0)	0 (0.0)	
Trabectedin/PLD	303 (99.7)	0 (0.0)	0 (0.0)	1 (0.3)	0 (0.0)	0 (0.0)	1 (0.3)	
**4. Eye disorders**								0.787
Carboplatin/PLD	285 (96.9)	9 (3.1)	0 (0.0)	0 (0.0)	0 (0.0)	0 (0.0)	0 (0.0)	
Trabectedin/PLD	299 (98.4)	2 (0.7)	3 (1.0)	0 (0.0)	0 (0.0)	0 (0.0)	0 (0.0)	
**-4.3. Photopsia**								0.309
Carboplatin/PLD	293 (99.7)	1 (0.3)	0 (0.0)	0 (0.0)	0 (0.0)	0 (0.0)	0 (0.0)	
Trabectedin/PLD	304 (100)	0 (0.0)	0 (0.0)	0 (0.0)	0 (0.0)	0 (0.0)	0 (0.0)	
**-4.4. Vision blurred**								0.023
Carboplatin/PLD	289 (98.3)	5 (1.7)	0 (0.0)	0 (0.0)	0 (0.0)	0 (0.0)	0 (0.0)	
Trabectedin/PLD	304 (100)	0 (0.0)	0 (0.0)	0 (0.0)	0 (0.0)	0 (0.0)	0 (0.0)	
**-4.99. Other**								0.368
Carboplatin/PLD	290 (98.6)	4 (1.4)	0 (0.0)	0 (0.0)	0 (0.0)	0 (0.0)	0 (0.0)	
Trabectedin/PLD	299 (98.4)	2 (0.7)	3 (1.0)	0 (0.0)	0 (0.0)	0 (0.0)	0 (0.0)	
**5. Gastrointestinal disorders**								0.000
Carboplatin/PLD	146 (49.7)	66 (22.4)	61 (20.7)	20 (6.8)	1 (0.3)	0 (0.0)	21 (7.1)	
Trabectedin/PLD	116 (38.2)	60 (19.7)	75 (24.7)	51 (16.8)	2 (0.7)	0 (0.0)	53 (17.4)	
**-5.1. Abdominal discomfort**								0.681
Carboplatin/PLD	292 (99.3)	1 (0.3)	1 (0.3)	0 (0.0)	0 (0.0)	0 (0.0)	0 (0.0)	
Trabectedin/PLD	302 (99.3)	2 (0.7)	0 (0.0)	0 (0.0)	0 (0.0)	0 (0.0)	0 (0.0)	
**-5.10. Tongue ulceration**								0.309
Carboplatin/PLD	293 (99.7)	1 (0.3)	0 (0.0)	0 (0.0)	0 (0.0)	0 (0.0)	0 (0.0)	
Trabectedin/PLD	304 (100)	0 (0.0)	0 (0.0)	0 (0.0)	0 (0.0)	0 (0.0)	0 (0.0)	
**-5.11. Flatulence**								0.187
Carboplatin/PLD	294 (100)	0 (0.0)	0 (0.0)	0 (0.0)	0 (0.0)	0 (0.0)	0 (0.0)	
Trabectedin/PLD	302 (99.3)	1 (0.3)	1 (0.3)	0 (0.0)	0 (0.0)	0 (0.0)	0 (0.0)	
**-5.12. Gastritis**								0.506
Carboplatin/PLD	291 (99.0)	2 (0.7)	1 (0.3)	0 (0.0)	0 (0.0)	0 (0.0)	0 (0.0)	
Trabectedin/PLD	303 (99.7)	0 (0.0)	1 (0.3)	0 (0.0)	0 (0.0)	0 (0.0)	0 (0.0)	
**-5.13. Gastroesophageal reflux disease**								0.973
Carboplatin/PLD	292 (99.3)	2 (0.7)	0 (0.0)	0 (0.0)	0 (0.0)	0 (0.0)	0 (0.0)	
Trabectedin/PLD	302 (99.3)	2 (0.7)	0 (0.0)	0 (0.0)	0 (0.0)	0 (0.0)	0 (0.0)	
**-5.14. Gastrointestinal haemorrhage**								0.981
Carboplatin/PLD	293 (99.7)	0 (0.0)	0 (0.0)	1 (0.3)	0 (0.0)	0 (0.0)	1 (0.3)	
Trabectedin/PLD	303 (99.7)	0 (0.0)	0 (0.0)	1 (0.3)	0 (0.0)	0 (0.0)	1 (0.3)	
**-5.15. Gastrointestinal pain**								0.640
Carboplatin/PLD	293 (99.7)	0 (0.0)	0 (0.0)	1 (0.3)	0 (0.0)	0 (0.0)	1 (0.3)	
Trabectedin/PLD	300 (98.7)	3 (1.0)	1 (0.3)	0 (0.0)	0 (0.0)	0 (0.0)	0 (0.0)	
**-5.18. Haemorrhoids**								0.325
Carboplatin/PLD	294 (100)	0 (0.0)	0 (0.0)	0 (0.0)	0 (0.0)	0 (0.0)	0 (0.0)	
Trabectedin/PLD	303 (99.7)	1 (0.3)	0 (0.0)	0 (0.0)	0 (0.0)	0 (0.0)	0 (0.0)	

	G0 n (%)	G1 n (%)	G2 n (%)	G3 n (%)	G4 n (%)	G5 n (%)	G3+G4+G5 n (%)	Chi-squared for trend
**-5.19. Ileus**								0.309
Carboplatin/PLD	293 (99.7)	0 (0.0)	0 (0.0)	1 (0.3)	0 (0.0)	0 (0.0)	1 (0.3)	
Trabectedin/PLD	304 (100)	0 (0.0)	0 (0.0)	0 (0.0)	0 (0.0)	0 (0.0)	0 (0.0)	
**-5.2. Abdominal distension**								0.036
Carboplatin/PLD	294 (100)	0 (0.0)	0 (0.0)	0 (0.0)	0 (0.0)	0 (0.0)	0 (0.0)	
Trabectedin/PLD	299 (98.4)	4 (1.3)	1 (0.3)	0 (0.0)	0 (0.0)	0 (0.0)	0 (0.0)	
**-5.23. Nausea**								0.000
Carboplatin/PLD	199 (67.7)	53 (18.0)	35 (11.9)	7 (2.4)	0 (0.0)	0 (0.0)	7 (2.4)	
Trabectedin/PLD	165 (54.3)	59 (19.4)	52 (17.1)	27 (8.9)	1 (0.3)	0 (0.0)	28 (9.2)	
**-5.28. Stomatitis**								0.025
Carboplatin/PLD	265 (90.1)	18 (6.1)	9 (3.1)	2 (0.7)	0 (0.0)	0 (0.0)	2 (0.7)	
Trabectedin/PLD	262 (86.2)	16 (5.3)	18 (5.9)	7 (2.3)	1 (0.3)	0 (0.0)	8 (2.6)	
**-5.29. Subileus**								0.172
Carboplatin/PLD	294 (100)	0 (0.0)	0 (0.0)	0 (0.0)	0 (0.0)	0 (0.0)	0 (0.0)	
Trabectedin/PLD	302 (99.3)	0 (0.0)	1 (0.3)	1 (0.3)	0 (0.0)	0 (0.0)	1 (0.3)	
**-5.30. Vomiting**								0.001
Carboplatin/PLD	242 (82.3)	30 (10.2)	17 (5.8)	4 (1.4)	1 (0.3)	0 (0.0)	5 (1.7)	
Trabectedin/PLD	217 (71.4)	43 (14.1)	24 (7.9)	20 (6.6)	0 (0.0)	0 (0.0)	20 (6.6)	
**-5.4. Abdominal pain**								0.269
Carboplatin/PLD	283 (96.3)	5 (1.7)	4 (1.4)	2 (0.7)	0 (0.0)	0 (0.0)	2 (0.7)	
Trabectedin/PLD	286 (94.1)	10 (3.3)	3 (1.0)	5 (1.6)	0 (0.0)	0 (0.0)	5 (1.6)	
**-5.6. Constipation**								0.131
Carboplatin/PLD	249 (84.7)	28 (9.5)	16 (5.4)	1 (0.3)	0 (0.0)	0 (0.0)	1 (0.3)	
Trabectedin/PLD	241 (79.3)	41 (13.5)	20 (6.6)	2 (0.7)	0 (0.0)	0 (0.0)	2 (0.7)	
**-5.7. Diarrhoea**								0.441
Carboplatin/PLD	259 (88.1)	21 (7.1)	10 (3.4)	4 (1.4)	0 (0.0)	0 (0.0)	4 (1.4)	
Trabectedin/PLD	271 (89.1)	24 (7.9)	6 (2.0)	3 (1.0)	0 (0.0)	0 (0.0)	3 (1.0)	
**-5.8. Dyspepsia**								0.416
Carboplatin/PLD	287 (97.6)	5 (1.7)	2 (0.7)	0 (0.0)	0 (0.0)	0 (0.0)	0 (0.0)	
Trabectedin/PLD	293 (96.4)	8 (2.6)	3 (1.0)	0 (0.0)	0 (0.0)	0 (0.0)	0 (0.0)	
**-5.9. Dysphagia**								0.583
Carboplatin/PLD	293 (99.7)	1 (0.3)	0 (0.0)	0 (0.0)	0 (0.0)	0 (0.0)	0 (0.0)	
Trabectedin/PLD	302 (99.3)	2 (0.7)	0 (0.0)	0 (0.0)	0 (0.0)	0 (0.0)	0 (0.0)	
**-5.99. Other**								0.743
Carboplatin/PLD	285 (96.9)	4 (1.4)	5 (1.7)	0 (0.0)	0 (0.0)	0 (0.0)	0 (0.0)	
Trabectedin/PLD	295 (97.0)	2 (0.7)	6 (2.0)	1 (0.3)	0 (0.0)	0 (0.0)	1 (0.3)	
**6. General disorders and administration site conditions**								0.003
Carboplatin/PLD	164 (55.8)	66 (22.4)	51 (17.3)	12 (4.1)	0 (0.0)	1 (0.3)	13 (4.4)	
Trabectedin/PLD	146 (48.0)	60 (19.7)	66 (21.7)	29 (9.5)	3 (1.0)	0 (0.0)	32 (10.5)	
**-6.1. Asthenia**								0.031
Carboplatin/PLD	244 (83.0)	23 (7.8)	26 (8.8)	1 (0.3)	0 (0.0)	0 (0.0)	1 (0.3)	
Trabectedin/PLD	238 (78.3)	23 (7.6)	33 (10.9)	9 (3.0)	1 (0.3)	0 (0.0)	10 (3.3)	
**-6.10. Fatigue**								0.538
Carboplatin/PLD	229 (77.9)	36 (12.2)	22 (7.5)	7 (2.4)	0 (0.0)	0 (0.0)	7 (2.4)	

	G0 n (%)	G1 n (%)	G2 n (%)	G3 n (%)	G4 n (%)	G5 n (%)	G3+G4+G5 n (%)	Chi-squared for trend
Trabectedin/PLD	236 (77.6)	30 (9.9)	28 (9.2)	10 (3.3)	0 (0.0)	0 (0.0)	10 (3.3)	
**-6.10. Sensation of foreign body**								0.309
Carboplatin/PLD	293 (99.7)	1 (0.3)	0 (0.0)	0 (0.0)	0 (0.0)	0 (0.0)	0 (0.0)	
Trabectedin/PLD	304 (100)	0 (0.0)	0 (0.0)	0 (0.0)	0 (0.0)	0 (0.0)	0 (0.0)	
**-6.12. Influenza like illness**								0.429
Carboplatin/PLD	293 (99.7)	1 (0.3)	0 (0.0)	0 (0.0)	0 (0.0)	0 (0.0)	0 (0.0)	
Trabectedin/PLD	302 (99.3)	1 (0.3)	1 (0.3)	0 (0.0)	0 (0.0)	0 (0.0)	0 (0.0)	
**-6.14. Malaise**								0.216
Carboplatin/PLD	293 (99.7)	1 (0.3)	0 (0.0)	0 (0.0)	0 (0.0)	0 (0.0)	0 (0.0)	
Trabectedin/PLD	301 (99.0)	1 (0.3)	2 (0.7)	0 (0.0)	0 (0.0)	0 (0.0)	0 (0.0)	
**-6.16. Mucosal inflammation**								0.470
Carboplatin/PLD	269 (91.5)	11 (3.7)	12 (4.1)	2 (0.7)	0 (0.0)	0 (0.0)	2 (0.7)	
Trabectedin/PLD	273 (89.8)	17 (5.6)	7 (2.3)	7 (2.3)	0 (0.0)	0 (0.0)	7 (2.3)	
**-6.18. Oedema**								0.036
Carboplatin/PLD	290 (98.6)	4 (1.4)	0 (0.0)	0 (0.0)	0 (0.0)	0 (0.0)	0 (0.0)	
Trabectedin/PLD	292 (96.1)	10 (3.3)	2 (0.7)	0 (0.0)	0 (0.0)	0 (0.0)	0 (0.0)	
**-6.19. Pain**								0.006
Carboplatin/PLD	292 (99.3)	1 (0.3)	0 (0.0)	1 (0.3)	0 (0.0)	0 (0.0)	1 (0.3)	
Trabectedin/PLD	288 (94.7)	9 (3.0)	7 (2.3)	0 (0.0)	0 (0.0)	0 (0.0)	0 (0.0)	
**-6.20. Pyrexia**								0.000
Carboplatin/PLD	289 (98.3)	5 (1.7)	0 (0.0)	0 (0.0)	0 (0.0)	0 (0.0)	0 (0.0)	
Trabectedin/PLD	280 (92.1)	14 (4.6)	6 (2.0)	2 (0.7)	2 (0.7)	0 (0.0)	4 (1.3)	
**-6.21. Swelling**								0.671
Carboplatin/PLD	293 (99.7)	1 (0.3)	0 (0.0)	0 (0.0)	0 (0.0)	0 (0.0)	0 (0.0)	
Trabectedin/PLD	303 (99.7)	0 (0.0)	1 (0.3)	0 (0.0)	0 (0.0)	0 (0.0)	0 (0.0)	
**-6.5. Catheter site pain**								0.325
Carboplatin/PLD	294 (100)	0 (0.0)	0 (0.0)	0 (0.0)	0 (0.0)	0 (0.0)	0 (0.0)	
Trabectedin/PLD	303 (99.7)	0 (0.0)	1 (0.3)	0 (0.0)	0 (0.0)	0 (0.0)	0 (0.0)	
**-6.8. Discomfort**								0.671
Carboplatin/PLD	293 (99.7)	1 (0.3)	0 (0.0)	0 (0.0)	0 (0.0)	0 (0.0)	0 (0.0)	
Trabectedin/PLD	303 (99.7)	0 (0.0)	1 (0.3)	0 (0.0)	0 (0.0)	0 (0.0)	0 (0.0)	
**-6.9. Extravasation**								0.325
Carboplatin/PLD	294 (100)	0 (0.0)	0 (0.0)	0 (0.0)	0 (0.0)	0 (0.0)	0 (0.0)	
Trabectedin/PLD	303 (99.7)	0 (0.0)	1 (0.3)	0 (0.0)	0 (0.0)	0 (0.0)	0 (0.0)	
**-6.99. Other**								0.930
Carboplatin/PLD	291 (99.0)	1 (0.3)	0 (0.0)	1 (0.3)	0 (0.0)	1 (0.3)	2 (0.7)	
Trabectedin/PLD	300 (98.7)	0 (0.0)	2 (0.7)	2 (0.7)	0 (0.0)	0 (0.0)	2 (0.7)	
**-Not reported**								0.325
Carboplatin/PLD	294 (100)	0 (0.0)	0 (0.0)	0 (0.0)	0 (0.0)	0 (0.0)	0 (0.0)	
Trabectedin/PLD	303 (99.7)	1 (0.3)	0 (0.0)	0 (0.0)	0 (0.0)	0 (0.0)	0 (0.0)	
**7. Hepatobiliary disorders**								0.000
Carboplatin/PLD	276 (93.9)	13 (4.4)	3 (1.0)	2 (0.7)	0 (0.0)	0 (0.0)	2 (0.7)	
Trabectedin/PLD	215 (70.7)	11 (3.6)	19 (6.3)	49 (16.1)	10 (3.3)	0 (0.0)	59 (19.4)	
**-7.1. Cholestasis**								0.175

	G0 n (%)	G1 n (%)	G2 n (%)	G3 n (%)	G4 n (%)	G5 n (%)	G3+G4+G5 n (%)	Chi-squared for trend
Carboplatin/PLD	286 (97.3)	6 (2.0)	1 (0.3)	1 (0.3)	0 (0.0)	0 (0.0)	1 (0.3)	
Trabectedin/PLD	290 (95.4)	8 (2.6)	4 (1.3)	2 (0.7)	0 (0.0)	0 (0.0)	2 (0.7)	
**-7.4. Hepatotoxicity**								0.000
Carboplatin/PLD	278 (94.6)	11 (3.7)	3 (1.0)	2 (0.7)	0 (0.0)	0 (0.0)	2 (0.7)	
Trabectedin/PLD	218 (71.7)	9 (3.0)	19 (6.3)	48 (15.8)	10 (3.3)	0 (0.0)	58 (19.1)	
**-7.99. Other**								0.325
Carboplatin/PLD	294 (100)	0 (0.0)	0 (0.0)	0 (0.0)	0 (0.0)	0 (0.0)	0 (0.0)	
Trabectedin/PLD	303 (99.7)	0 (0.0)	0 (0.0)	1 (0.3)	0 (0.0)	0 (0.0)	1 (0.3)	
**8. Immune system disorders**								0.151
Carboplatin/PLD	281 (95.6)	4 (1.4)	8 (2.7)	1 (0.3)	0 (0.0)	0 (0.0)	1 (0.3)	
Trabectedin/PLD	297 (97.7)	2 (0.7)	5 (1.6)	0 (0.0)	0 (0.0)	0 (0.0)	0 (0.0)	
**-8.3. Hypersensitivity**								0.151
Carboplatin/PLD	281 (95.6)	4 (1.4)	8 (2.7)	1 (0.3)	0 (0.0)	0 (0.0)	1 (0.3)	
Trabectedin/PLD	297 (97.7)	2 (0.7)	5 (1.6)	0 (0.0)	0 (0.0)	0 (0.0)	0 (0.0)	
**9. Infections and infestations**								0.003
Carboplatin/PLD	286 (97.3)	3 (1.0)	3 (1.0)	1 (0.3)	1 (0.3)	0 (0.0)	2 (0.7)	
Trabectedin/PLD	277 (91.1)	7 (2.3)	13 (4.3)	5 (1.6)	1 (0.3)	1 (0.3)	7 (2.3)	
**-9.1. Candidiasis**								0.325
Carboplatin/PLD	294 (100)	0 (0.0)	0 (0.0)	0 (0.0)	0 (0.0)	0 (0.0)	0 (0.0)	
Trabectedin/PLD	303 (99.7)	0 (0.0)	1 (0.3)	0 (0.0)	0 (0.0)	0 (0.0)	0 (0.0)	
**-9.10. Pharyngitis**								0.325
Carboplatin/PLD	294 (100)	0 (0.0)	0 (0.0)	0 (0.0)	0 (0.0)	0 (0.0)	0 (0.0)	
Trabectedin/PLD	303 (99.7)	1 (0.3)	0 (0.0)	0 (0.0)	0 (0.0)	0 (0.0)	0 (0.0)	
**-9.11. Pneumonia**								0.164
Carboplatin/PLD	294 (100)	0 (0.0)	0 (0.0)	0 (0.0)	0 (0.0)	0 (0.0)	0 (0.0)	
Trabectedin/PLD	302 (99.3)	0 (0.0)	0 (0.0)	2 (0.7)	0 (0.0)	0 (0.0)	2 (0.7)	
**-9.12. Respiratory infection**								0.053
Carboplatin/PLD	294 (100)	0 (0.0)	0 (0.0)	0 (0.0)	0 (0.0)	0 (0.0)	0 (0.0)	
Trabectedin/PLD	300 (98.7)	0 (0.0)	3 (1.0)	1 (0.3)	0 (0.0)	0 (0.0)	1 (0.3)	
**-9.13. Sepsis**								0.696
Carboplatin/PLD	292 (99.3)	1 (0.3)	0 (0.0)	0 (0.0)	1 (0.3)	0 (0.0)	1 (0.3)	
Trabectedin/PLD	302 (99.3)	0 (0.0)	0 (0.0)	1 (0.3)	0 (0.0)	1 (0.3)	2 (0.7)	
**-9.15. Skin infection**								0.325
Carboplatin/PLD	294 (100)	0 (0.0)	0 (0.0)	0 (0.0)	0 (0.0)	0 (0.0)	0 (0.0)	
Trabectedin/PLD	303 (99.7)	1 (0.3)	0 (0.0)	0 (0.0)	0 (0.0)	0 (0.0)	0 (0.0)	
**-9.17. Urinary tract infection**								0.255
Carboplatin/PLD	292 (99.3)	0 (0.0)	2 (0.7)	0 (0.0)	0 (0.0)	0 (0.0)	0 (0.0)	
Trabectedin/PLD	298 (98.0)	2 (0.7)	4 (1.3)	0 (0.0)	0 (0.0)	0 (0.0)	0 (0.0)	
**-9.2. Catheter site infection**								0.325
Carboplatin/PLD	294 (100)	0 (0.0)	0 (0.0)	0 (0.0)	0 (0.0)	0 (0.0)	0 (0.0)	
Trabectedin/PLD	303 (99.7)	1 (0.3)	0 (0.0)	0 (0.0)	0 (0.0)	0 (0.0)	0 (0.0)	
**-9.5. Herpes virus infection**								0.429
Carboplatin/PLD	293 (99.7)	1 (0.3)	0 (0.0)	0 (0.0)	0 (0.0)	0 (0.0)	0 (0.0)	
Trabectedin/PLD	302 (99.3)	1 (0.3)	1 (0.3)	0 (0.0)	0 (0.0)	0 (0.0)	0 (0.0)	

	G0<br>n (%)	G1<br>n (%)	G2<br>n (%)	G3<br>n (%)	G4<br>n (%)	G5<br>n (%)	G3+G4+G5<br>n (%)	Chi-squared for trend
**-9.6. Herpes zoster**								0.513
Carboplatin/PLD	293 (99.7)	0 (0.0)	0 (0.0)	1 (0.3)	0 (0.0)	0 (0.0)	1 (0.3)	
Trabectedin/PLD	303 (99.7)	1 (0.3)	0 (0.0)	0 (0.0)	0 (0.0)	0 (0.0)	0 (0.0)	
**-9.7. Infection**								0.254
Carboplatin/PLD	292 (99.3)	1 (0.3)	1 (0.3)	0 (0.0)	0 (0.0)	0 (0.0)	0 (0.0)	
Trabectedin/PLD	300 (98.7)	0 (0.0)	3 (1.0)	1 (0.3)	0 (0.0)	0 (0.0)	1 (0.3)	
**-9.8. Abscess**								0.325
Carboplatin/PLD	294 (100)	0 (0.0)	0 (0.0)	0 (0.0)	0 (0.0)	0 (0.0)	0 (0.0)	
Trabectedin/PLD	303 (99.7)	0 (0.0)	0 (0.0)	1 (0.3)	0 (0.0)	0 (0.0)	1 (0.3)	
**-9.99. Other**								0.180
Carboplatin/PLD	293 (99.7)	0 (0.0)	1 (0.3)	0 (0.0)	0 (0.0)	0 (0.0)	0 (0.0)	
Trabectedin/PLD	300 (98.7)	1 (0.3)	1 (0.3)	1 (0.3)	1 (0.3)	0 (0.0)	2 (0.7)	

Table s4. Quality of life - Change from baseline

	Change carboplatin/PLD Mean (SD)	Change trabectedin/PLD Mean (SD)	Difference trabectedin/PLD vs. carboplatin/PLD Mean (95%CI)
**QLQ-C30**	N=156	N=170	
Global health status /QoL	-4.4 (21.0)	-11.7 (25.4)	-7.3 (-12.4,-2.2)
**Functional scales**			
Physical functioning	-8.5 (20.0)	-12.9 (22.6)	-4.3 (-9.0,0.4)
Role functioning	-10.6 (27.1)	-15.1 (31.2)	-4.5 (-10.8,1.8)
Cognitive functioning	-3.5 (17.4)	-4.9 (17.2)	-1.5 (-5.2,2.2)
Emotional functioning	2.4 (22.4)	0.1 (23.1)	-2.4 (-7.3,2.5)
Social functioning	-6.8 (27.0)	-10.6 (24.8)	-3.8 (-9.5,1.9)
**Symptom scales/items**			
Fatigue	10.9 (23.2)	19.1 (25.6)	8.1 (2.8,13.4)
Nausea and vomiting	3.2 (23.0)	11.0 (25.2)	7.8 (2.5,13.1)
Pain	0.3 (26.6)	4.8 (27.5)	4.4 (-1.5,10.3)
Dyspnoea	6.5 (27.5)	11.1 (29.3)	4.6 (-1.7,10.9)
Insomnia	-0.2 (32.3)	-2.8 (27.9)	-2.6 (-9.3,4.1)
Appetite loss	2.6 (28.1)	15.2 (32.1)	12.6 (5.9,19.3)
Constipation	6.9 (31.0)	6.9 (31.6)	0 (-6.9,6.9)
Diarrhoea	4.1 (23.0)	0.6 (24.9)	-3.5 (-8.8,1.8)
Financial difficulties	1.5 (25.9)	4.2 (22.4)	2.7 (-2.6,8.0)
**QLQ-OV28**	N=159	N=168	
**Functional scales**			
Body immage	-3.1 (27.3)	-7.1 (29.2)	-3.9 (-10.2,2.4)
Sexuality	1.9 (18.6)	2.7 (19.7)	0.8 (-3.5,5.1)
Attitude to disease/treatment	0.6 (28.8)	-7.7 (25.5)	-8.4 (-14.3,-2.5)
**Symptom scales/items**			
Abdominal/GI	0.6 (17.6)	2.4 (15.6)	1.7 (-1.8,5.2)
Peripheral neuropathy	6.6 (23.0)	9.6 (23.5)	3 (-2.1,8.1)
Hormonal/ menopausal symptoms	-1.5 (21.8)	3.9 (26.6)	5.4 (0.1,10.7)
Other chemotherapy side effects	6.3 (17.7)	10.4 (16.2)	4 (0.3,7.7)
Hair loss	7.9 (23.8)	7.2 (28.2)	-0.6 (-6.3,5.1)

Legend: SD: stadard deviation. 95%CI: 95% Confidence Interval

Variable	Events/no. (%)	HR [95%CI]
**OVERALL**	471/611 (77.09)	1.13 [0.94-1.35]
**BRCA**		0.049*
Wild-type	231/290 (79.66)	1.02 [0.78-1.32]
Mutated	40/71 (56.34)	0.95 [0.51-1.78]
Not known	198/247 (80.16)	1.45 [1.09-1.92]
**Measurable disease at study entry**		0.247*
No	133/173 (76.88)	0.95 [0.68-1.34]
Yes	338/438 (77.17)	1.21 [0.98-1.50]
**Previous treatment with anthracycline**		0.532*
No	426/556 (76.62)	1.14 [0.95-1.38]
Yes	45/55 (81.82)	0.90 [0.50-1.62]
**Number of prior treatments**		0.071*
1	332/425 (78.12)	1.25 [1.01-1.55]
2	138/185 (74.59)	0.87 [0.62-1.22]
**Size of residual disease after initial surgery**		0.701*
<=1 cm	242/316 (76.58)	1.03 [0.80-1.33]
>1 cm	111/146 (76.03)	1.42 [0.98-2.07]
Not known	110/141 (78.01)	1.04 [0.71-1.51]
**Histology**		0.622*
Serous	405/517 (78.34)	1.15 [0.95-1.40]
Not serous	66/94 (70.21)	1.00 [0.62-1.62]
**Patients receiving subsequent therapy**		0.142*
No	112/121 (92.56)	1.38 [0.95-2.01]
Yes	352/452 (77.88)	1.08 [0.88-1.33]
-Yes – platinum therapy (exp arm)	330/423 (78.01)	1.06 [0.85-1.32]

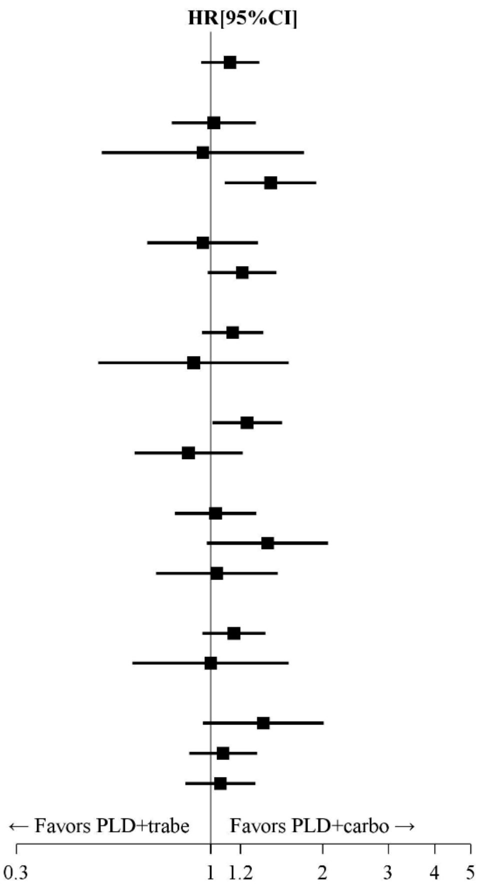

* interaction test

Figure s1. Forest plot of overall survival by subgroups

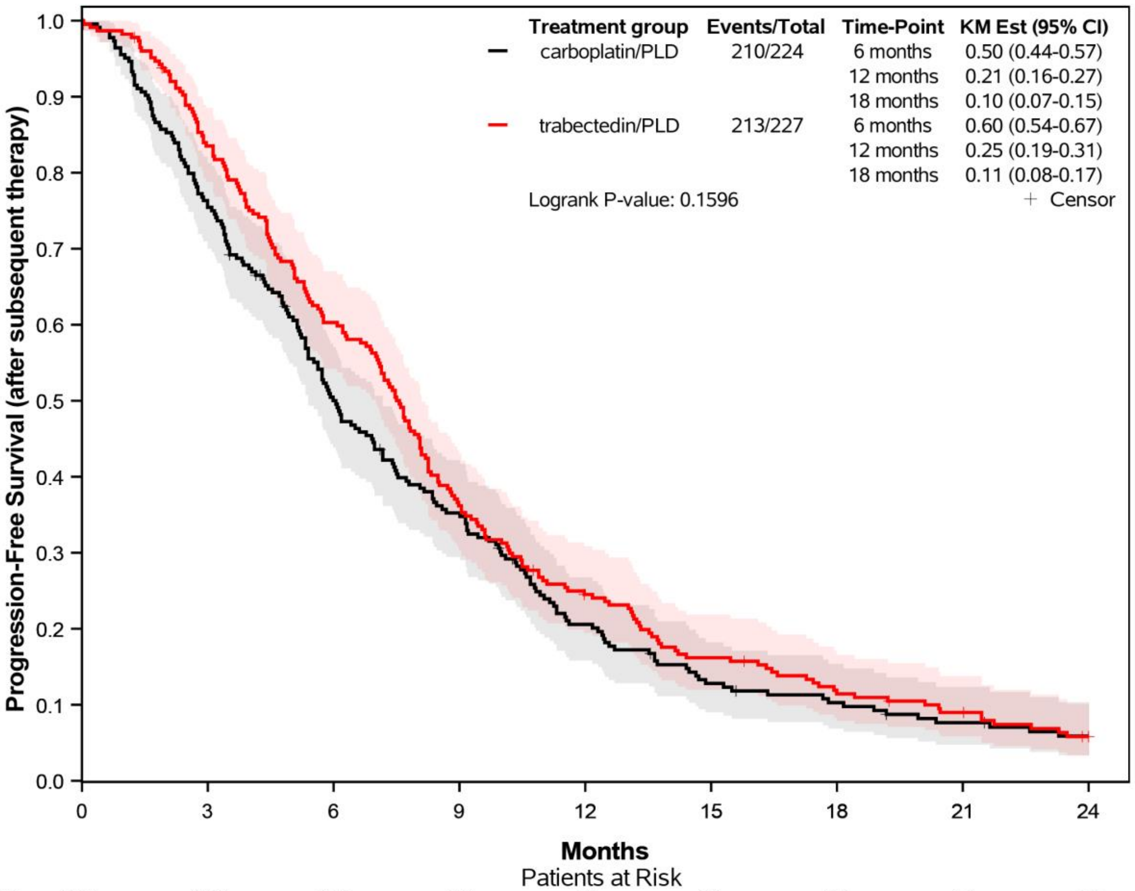

Patients at Risk

carboplatin/PLD	224	169	109	76	43	26	20	14	10
trabectedin/PLD	227	187	135	81	53	35	24	18	10

Figure s2. Kaplan-Meier curves for progression-free survival after subsequent therapy (PFS-ST)

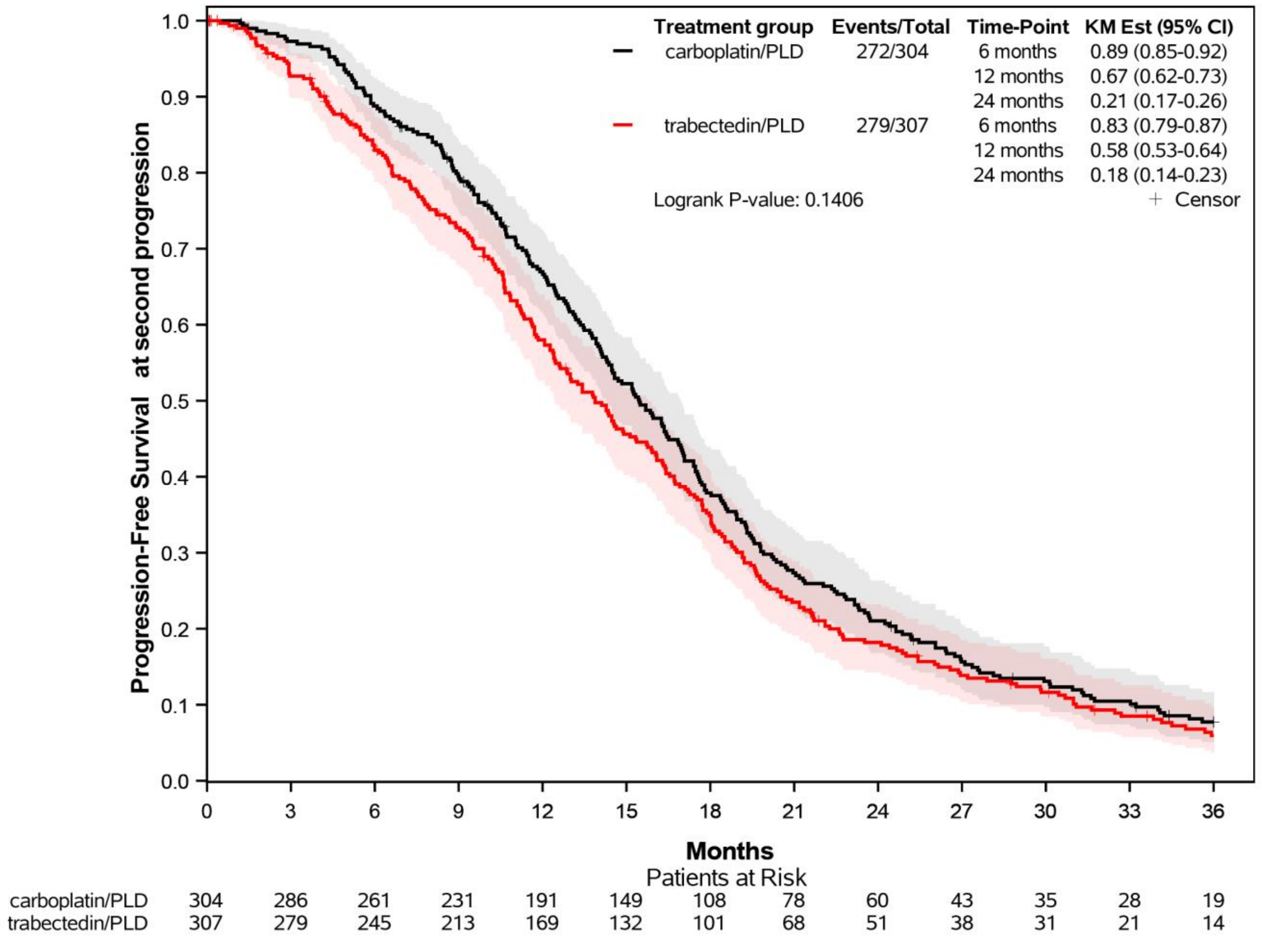

Figure s3. Kaplan-Meier curves for progression-free survival at the second progression (PFS2)

Appendix A - INOVATYON study group

Study Sponsor: Elena Biagioli[4], Roldano Fossati[4], Eliana Rulli[4], Giuseppe Funari[4], Luciano Carlucci[4], Davide Poli[4], Paola Tonto[4], Fabio Galli[4], Maria Clara Caudana[4], Chiara Carpentieri[4], Elda Caccia[4], Francesca Galli[4].

Mario Negri Gynecologic Oncology group-MaNGO (MaNGO)

Nicoletta Colombo[1], Mariateresa Lapresa[36], Gabriella Parma[36], Abbondanza Montinaro[36], Rita Liuzzi[36], Caterina Parillo[36], Angiolo Gadducci[2], Stefania Cosio[2], Giulia Tasca[20], Maria Ornella Nicoletto[20], Alessandra Baldoni[20], Giulia Doria[20], Giulietta Sinigaglia[20], Germana Tognon[22], Eliana Bignotti[22], Martina Ratti[22], Monica Ragnoli[22], Valentina Zizioli[22], Laura Zanotti[22], Rossella Franzini[22], Andrea De Censi[24], Irene Maria Briata[24], Silvia Giuliano[24], Paola Zola[26], Dionyssios Katsaros[26], Fulvio Borella[26], Emilie Marion Canuto[26], Maria Elena Laudani[26], Martina Barboni[26], Elisa Piovano[26], Elena Peirano[26], Pierluigi Benedetti Panici[27], Innocenza Palaia[27], Federica Tomao[27], Serena Boccia[27], Claudia Marchetti[27], Massimo Aglietta[28], Giorgio Valabrega[46], Eleonora Geninatti[46], Cosimo Martino[46], Marco Bonaudo[46],Valentina Arcangeli[29], Giuliana Drudi[29], Claudio Zamagni[30], Cinzia Pizzirani[30], Daniela Rubino[30], Alessandra Bologna[31], Annalisa Berselli[31], Erika Gervasi[31], Roberta Gnoni[31], Alessandro Bertolini[37], Elisabetta Menatti[37], Cinzia Caroti[38], Milena Bruzzone[38], Maurizio Cosso[38], Barbara Rebesco[38], Nicoletta Donadello[39], Chiara Apolloni[39],Gianna Di Costanzo[40], Paola Curcio[40], Francesco Giurlanda[40], Silvia Violante[40], Alberto Zaniboni[41], Brunella Di Biasi[41], Laura Zanotti[41], Daniela Surico[42], Roberta Buosi[42], Paola Maggiora[42], Gloria Borra[42], Stefania Cresta[42], Enrico Cortesi[43], Alessandra Gargiulo[43], Sofia Verkhouskaia[43], Elena Zafarana[44], Francesca Del Monte[44], Vittorio Fusco[45], Iolanda De Martino[45], Lorenza Randi[45], Giovanna Bellotti[45], Laura Zavallone[47], Elisa Perfetti[47], Emanuela Reale[47], Barbara Sartorello[47], Mario Clerico[47], Teresa Gamucci[48], Filomena Narducci[48], Silvia Quadrini[48], Valentina Musacchi[49], Luciana Babilonti[49], Chiara Cassani[49], Annamaria Ferrero[50], Daniela Attianese[50], Isabella Cipullo[50], Michela Villa[50], Enrico Badellino[50], Elisa Tripodi[50], Valentina Zanfagnin[50], Luigi Cavanna[51], Camilla Di Nunzio[51], Claudia Biasini[51], Livia Bidin[51], Roberto Sabbatini[52], Laura Casolari[52], Elisa Pettorelli[52], Ugo De Giorgi[25], Monia Dall'Agata[25], Giorgia Ravaglia[25], Stefano Tamberi[53], Alessandra Piancastelli[53], Maria Rosa Gentili[54], Enrico Campadelli[54], Claudia Casanova[54], Alessandra Piancastelli[54], Grazia Artioli[55], Silvia Coccato[55], Antonio Ardizzoia[56], Cristina Corbetta[56], Carlo Curioni[56], Alessandra Crippa[56], Federica Villa[56], Davide Pellizzoni[56], Alessia Caldara[57], Renza Triolo[57], Zuzana Sirotovà[58], Giulia Courthod[58], Cristina Barè[58], Clelia Casartelli[59], Anna Tusei[59], Deborah Battagin[59], Michele Aieta[60], Antonella Lapenta[60], Saverio Cinieri[61], Margherita Cinefra[61], Enrica Mazzoni[61], Gabriella Del Bene[61], Maria D'Amico[61], Elvira De Marino[62], Micol Caccianotti[62], Stefania Gori[63], Fabiana Marchetti[63], Francesco Ferraù[64], Claudia Masi[64], Giovanna Antonelli[64], Livio Blasi[65], Massimiliano Alù[65], Sabino De Placido[66], Rossella Luria[66], Cinzia Cardalesi[66], Annalisa Ilardi[66], Carlo Milandri[67], Giuditta Zipoli[67].

Grupo Español de Investigación en Cáncer de Ovario (GEICO)

Cristina Churruca Galaz[11], Maria Diez Zubizarreta[11], Ana Paisán Ruiz[11], Koro Andueza Granados[11], Nerea Ancizar Lizarraga[11], Isabel Alvarez Lopez[11], Maria Pilar Barretina[15], Mertitxell Rigola[15], Francesca Llopis[15], Blanca Martinez[15], Begoña Martin[15], Samiha Saidani[15], Isabel Bover[18], Juan Coves[18], Sabina Soler[18], Sergio Ayuga[18], Maria Iglesias[18], Margarita Romeo[19], Diana Roa[19], Jose Luis Cuadra[19], Isabel Gomez[19], Judith Rodriguez Moreno[19], Christian Querol Orgaz[19], Eugenia López Sanchez[19], Beatriz Pardo[21], Marta Pamias Noguè[21], Marta Gil Martín[21], Jokin Yllera Retegui[21], Maria Jesus Rubio[23], Maria Jose Marín[23], Manuel Rejano Castaneda[23], Juan Parras[23], Rebecca Pavon Gomez[23], Maria Pilar Mesa Blanco[23], Andrés Poveda[35], Ignacio Romero[35], Gema Piqueres[35], Maria Muntaner[35], Pedro Mallol[35], Carlos Peris[35], Ana Santaballa[68], Laura Palomar[68], Helena de la Cueva[68], Marian Lavin[68], Raúl Márquez[69], Antonio Gonzalez Martin[69], Raquel Bratos[69], Marta Gutierrez[69], Ana Cortijo[69], Jesus Alarcon[70], Aranzazu Gonzalez Del Alba[70], Antonia Perello[70], Marga Oliver[70], Cristina Caballero Diaz[71], Ana Blasco[71], Virginia Palomar[71], Francisco García[71], Rosa Monfort[71], Vicente Castellano[71], Teresa Fernandez[71], Nuria Ruiz Miravet[72], Regina Romero[72], Llorens, Kavita Gandhi Morar[72], Francisco Garcia Pinon[72], Javier Munarriz Ferrandis[72], Gema Gimeno Mateu[72], Eugenia Ortega[73], Ruben Tejero[73], Marina Pardina[73], Laura Runi Carrera[73], Angels Arcusa Lanza[74], Carme Aracil[74], Luis Fernandez[74], Meritxell Vilanova[74], Nuria Barragan[74], Manuela Gonzalez Navarro[74], Silvia Catot Tort[75], Alba Tubau Rosell[75], Mireia Porquet Prat[75], Irene Oller Sales[75], Elena Garcia Martinez[76], Jose Antonio Lopez Oliva[76], Elisa Garcia Garre[76], Francisco Ayala de la Pena[76], M.Dolores Najera Perez[76], Jumia Pina Mingorance[76], Regina Girones[77], Carmen Salvador[77], Isabel Navarro Cortina[77], M Angeles Lopez Montenegro Soria[77], Elisa Marti Beneito[77], Yolanda Garcia[78], Mercé Abad[78], Miquel Cruel[78], Irene López[78], Evelyn Herrera[78], Cesar Mendiola[79], Luis Manso, Rosa Rodriguez[79], Ana Beatriz Sanchez[80], Teresa Quintanar[80], Rosana Perea[80], Eric Campello[80], Elena Garcia Martinez[81], Jose Antonio Lopez Oliva[81], Elisa Garcia Garre[81], Francisco Ayala de la Pena[81], M.Dolores Najera Perez[81], Jumia Pina Mingorance[81].

North-Eastern-German Society of Gynaecological Oncology (NOGGO)

Jalid Sehouli[3], Radoslav Chekerov[3], Mustafa Deryal[16], Mira Ebner[16], Kathrin Alt[16], Ulrike Schilling[16], Pauline Wimberger[34], Ulrich Canzler[86], Christine Leffler[86], Christine Griebsch[86], Bettina Epperlein[86], Karolin Franke[86], Helen Urban[86], Helen Krieger[86], Elena Ioana Braicu[82], Nils Leiminger[82], Isil Yalcinkaya[82], Georg Heinrich[83], Christiane Richter-Ehrenstein[83], Evelyn Mende[83], Cindy Drewitz[83], Ingo Runnebaum[84], Matthias Rengsberger[84], Jorge Jimenez Cruz[84], Marc Radosa[84], Beate Ludwig[84], Sven Mahner[85], Volkmar Müller[85], Natasha Gaskill[85], Natasha Gaskill[85], Esther Freese[85], Sandra Bertram-Schemmel[85], Sylke Krenkel[85], Barbara Schmalfeldt[85], Fabian Trillsch[85], Gülten Oskay-Özcelik[87], Hans-Joachim Hindenburg[87], Simone Diepold[87], Gülizar Yurtseven[87], Angret Wetzel[87], Maike de Wit[88], Gerd Büschel[88], Dorothea Scholz[88], Winfried Rothaug[88], Gersbacher-Runge[88], Eva-Maria Grischke[89], Christian Gall[89], Emily Irslinger[89], Yvonne Löffler[89], Eva Stauß[89], Dirk Bauerschlag[90], Nadine Sallach[90], Gesa Voss[90], Antje Hempel[90], Christian Schem[90], Florian Heitz[91], Philipp Harter[91], Andreas du Bois[91], Claudia Nölling[91], Anna Zarebski[91], Belinda Borrmann[91], Karin Hellmann[91], Alexander Mustea[92], Dominique Koensgen- Mustea[92], Franziska Schindler[92], Katharina Stips[92], Antje Belau[92], Tanja Fehm[93], Markus Fleisch[93], Melanie Kruppa[93], Astrid Grosssteinbeck[93], Ulrike Kreimer[93], Thomas Kaleta[93], Karin Reichert[93], Melvy Baum[93], Eugen Ruckhäberle[93], Andrea Heider[94], Dagmar Sent[94], Vera Heidrich[94], Jeanette Zimmermann[94], Max Dieterich[95], Juliane Terpe[95], Beatrice Engel[95], Michaela Stecher[95], Martina Groop-Meier[96], Thomas Decker[96], Irina Gossen[96], Christina Strobel[96],Marco Battista[97], Marcus Schmidt[97], Martina Seehase[97], Bettina Al-Kadi[97], Achim Woeckel[98], Mathias Krockenberger[98], Daniel Herr[98], Elisabeth Thees[98], Katrin Erdmann[98], Ivo Meinhold-Heerlein[99], Tomas Kupec[99], Julia Serno[99], Gertrud Strauch[99].

NHS National Services Scotland

Ana Montes[7], Malahat Khaul[7], Lisa Ng[7], Shifa Belul[7], Abhijit Jadhav[7], Agnieska Zielonka[7], Veronica D'Alessandro[7], Sara Lombardi[7], Rebecca Herbertson[14], Isobelle Coombes[14], Catherine Hunter[14], Emily Peasgood[14], Sara Hawkins[14], Kirsty Bracewell[14], Sara Hawkins[100], Carolyn Noren[100], Raquel Marcos Gomez[100], Tan Tsawayo[100], Jeanette Gilbert[100], Marian Flynn-Batham[100], Rachel Jones[101], Charlotte Young[101], Amanda Jackson[101], Kathy Bishop[101], Chaterine Watkins[101], Jayne Richards[101], Emma Hudson[101], Loiuse Hanna[101], Anila Parveen[101] Rebecca Bowen[102], Samantha Curtis[102], Thomas Tylee[102], Tania Allen[102], Michael Daly[102].

Belgian Gynaecological Oncology Group (BGOG)

Lionel D'Hondt[103], Geraldine Mineur[103], Cossette Sanchez[103], Frederic Bellemans[103], Ignace Vergote[10], Leen Trappeniers[104], Daisy Supply[104], Marijke Gaekens[104], Peter Vuylsteke[105], Dominique Crasson[105], Stéphanie Henry[105], Caroline Yague Sanz[105], Christof Vulsteke[106], An Casneuf[106], Iris Kyle-Leinhase[106], Mieke Van Den Berghe[106].

Dutch Gynaecological Oncology Group (DGOG)

Petronella Beatrix Ottevanger[8], Elvira Spoelman-Hecker[8], Claudia vanKerkhof-Peters[8], Anita Huisman[8], Geert Jan van der Goor[8], AM Westermann[32], Lyda ter Hofstede[32], E.H. Van Daalen[32], Fioole Lisanne[32], Frank Abe Nijenhuis[32], J.M. Tromp[32].

Swiss Group for Clinical Cancer Research (SAKK)

Cristiana Sessa[6], Giancarla Busi[6], Sheila Gaggetta[6], Anastasios Stathis[6], Gaia Griguolo[6], Sabine Van Den Bosch[6], Laura Moser[6], Massimiliano Salati[6], Lorenzo Rossi[6], Marco Pasqualotto[6], Luca Canziani[6], Vanessa Escudero[6], Marco Maria Aiello[6], Barbara Muoio[6], Mara Mantiero[6], Chiara Cerrato[6], Lorenzo Baldecchi[6], Juliana Datrocinio Ferreira[6], Salome Riniker[13], Brigitte Künzle[13], Anja Harder[13], Patrik Weder[13], Ursula Hasler-Strub[13], Thomas Ruhstaller[13], Belinda Germann[13], Beat Thürlimann[13], Stefan Diem[13], Ulrich Güller[13], Ruth Demmer[13], Jeanine Kehl[13], Bogumila Pfister[13], Nanda Meister[13], Sabine Schmid[13], Lotus Desbiolles[13], Aurelius Omlin[13], Christina Appenzeller[13], Stefanie Fischer[13], Patrizia Niedermann[13], Angelika Bickel[13], Claudio Thurneysen[13], Viola Heinzelmann[33], Marcus Vetter[33], Doris Weber[33], Ute Heisner[33], Mirjam Maier[33], Andrea Junghans[33], Edward Wignt[33], Martina Machacek[33], Fabienne Schwab[33], Brigitta Kautz[33], Rozolita Sejdini[33], Roger Von Moos[107], Franziska Hellmann[107], Gillian Roberts[107], Michael Mark[107], Sara Bastian[107], Gabriela Manetsch[107], Radmila Moudry[107], Karin Hohloch[107], Sabrina Zortea[107], Michael Schwitter[107], Tobias Freyholdt[107], Gisela Mayer[107], Alexandra Jori[107], Nadine Kowel-Beh[107], Katrin Oppel[107], Stefan Greuter[107], Elena Kralidis[108], Elias Widmer[108], Sabine Hunziker[108], Stephanie Aaltola[108], Christoph Mamot[108], Peter Moosmann[108], Alexander Schreiber[108], Angela Wolf[108], Ramona Olivia Merki[108], Wolf-Dieter Janthur[108], Ralph Zachariah-Gerber[108], Therese Gurtner[108], Julia Püschel[108], Stefanie Hetzenecker[108], Tobias Wehrhahn[108], Yvonne Meier[108], Katrin Schmutz[108], Anna Nowicka[108], Constanze Spelters[108], Denise Marini[108], Michael Mueller[109], Juliette Wanner[109], Manuela Rabaglio[109], Urban Novak[109], Veronika Pasquinelli[109], Martin Zweifel[109], Silvia Imboden[109], Y. Chittzzhatun Keveian[109], Barbara Rohrbach[109], Stefan Aebi[110], Stephanie Largiadèr[110], Evelyn Rickenbacher[110], Ralph Winterhalder[110], Marcel Zefferer[110], Violetta Powajbo[110], Daniela Weiler[110], Catrina Uhlmann Nussbaum[111], Walter Mingrone[111], Manuel Jungi[111], Therese Gurtner[111], Monique Hartmann[111], Céline Stäuble[111], Francina Hartmann[111], Mathias Fehr[112], Patricia Städele[112], Joellen Welter[112], Lea Köchli[112], Regula Jung[112], Wieland Sell[112], Alexander Scheffzek[112], Nicole Homburg[112], Mareike

Göranson[112], Birgit Luther[112], Andreas Müller[113], Beatrice Brinkers[113], Martina Pfitzner[113], Miklos Pless[113], Melanie Löffler[113], Natalie Fischer[113], Martin Kaelin[113], Sabina Schacher[113], Saskia Herrlich[113], Laetitia Mauti[113], Christian Taverna[114], Susan Baumann[114], Roman Inauen[114], Christina Schneider Taverna[114], Katrin Furrer[114], Franziska Borer[114], Sonja Burkhalter[114], Sarah Reich[114], Blanca Brandes[114], Rahel Hein[114], Barbara Heinzer[114], Annette Park[114], Simone Schlegel[114], Julia Schmidschneider[114], Rita Tast[114], Elisabeth Thalmann Fauser[114], Evelin Veres[114].

Nordic Society of Gynaecological Oncology (NSGO)

National PI: Mansoor Raza Mirza[17]

Johanna Mäenpää[5], Sami Saarelainen[5], Maarit Vuento[5], Susanna Honkonen[5], Heidi Kiiskinen[5], Gitte-Bettina Nyvang[12], Annemieke Sibtsen[12], Tina Vinsten Clausen[12],Gunnar B. Kristensen[115], Hege L Rasmussen[115], Iselin B. Broen[115], Karin Skogfjord[115], Per Arne Stensager[115], Anne Gry Bentzen[116], Gunn-Janne Paulsen[116], Kristin Iren Jensen[116], Anne Beate Vereide[116], Bent Fiane[117], Aase Merete Thuen[117], Tove Buhl[117], Ulla Puistola[118], Paivi Marttila[118], Paula Vaisanen[118], Marja Simojoki[118], Marianne Hinkula[118], Maarit Anttila[119], Marja Komulainen[119], Elina Puumalainen[119], Helena Oksa[119], Marjukka Sallien[119], Jatta Jokinen[119], Heli Riepponen[119], Heli Leinonen[119].

AGO Studienzentrale (AGO-Austria)

Christian Marth[9], Alain Zeimet[9], Daniel Reimer[9], Jasmina Suljkanovic[9], Regina Berger[9], Angela Riha[9], Irina Tsibulak[9], Edgar Petru[120], Hermann Fuchs[120], Birgit Wunsch[120], Angelika Lanz-Veit[120], Herta Schaffernak[120], Christoph Benedicic[120], Christian Schauer[121], Barbara Herritsch[121], Gabriele Moitzi[121], Carmen Siebenhofer[121], Bettina Werlitsch[121], Patrick Greimel[121], Alexander Reinthaller[122], Stephan Polterauer[122], Franziska Riedl[122], Christoph Grimm[122], Stefanie Aust[122], Veronika Seebacher[122], Richard Schwameis[122].

National coordinators

Marianne Linkels[8], Regina Berger[9], Angela Riha[9], Kirsten Murray[123], Jackie Burns[123], Ana Levin[124], Maren Keller[125], Simone Wyss[126], Michaela Werfeli[126], Line Seierøe Ulbæk[127], Sanne Lindberg[127].

1 Gynecologic Cancer Program, European Institute of Oncology IRCCS and University of Milan-Bicocca, Milan, Italy.2 Clinical and Experimental Medicine, Division of Gynecology and Obstetrics, University of Pisa, Pisa, Italy. 3 NOGGO Study group and Gynecology with Center for Oncological Surgery, European Competence Center for Ovarian Cancer, Charité-Berlin University of Medicine, Berlin, Germany 4 Istituto di Ricerche Farmacologiche Mario Negri IRCCS, Milano, Italy. 5 NSGO-CTU & Department of Obstetrics and Gynaecology and Tays Cancer Centre Tampere University and University Hospital, Tampere, Finland .6 Istituto Oncologico della Svizzera Italiana, IOSI, Bellinzona, Switzerland and SAKK .7 Guys and St Thomas' NHS Foundation Trust, London, UK. 8 Medical Oncology, Radboud University Medical Center, Nijmegen, Netherlands. 9 Medical University of Innsbruck, Department for Gynecology and Obstetrics, Innsbruck, Austria, and AGO Austria 10 Gynecologic Oncology, University Hospitals Leuven, Leuven Cancer Institute, and BGOG, Leuven, Belgium, European Union .11 Hospital de Donosti San Sebastián, Spain and GEICO. 12 NSGO-CTU & Department of Oncology, Odense University Hospital, Odense, Denmark. 13 Oncology, Kantonsspital St. Gallen, St. Gallen, Switzerland and SAKK .14 Brighton and Sussex University Hospitals NHS Trust, Brighton UK. 15 Institut Catalá de Oncología de Girona, Spain and GEICO.16 NOGGO Study group and CaritasKlinikum St. Theresia – Saarbruecken, Germany. 17 NSGO-CTU & Department of Cancer Treatment, Copenhagen University Hospital, Copenhagen, Denmark 18 Hospital Son Llatzer, Palma de Mallorca, Spain.19 Institut Catalá de Oncología de Badalona, Spain and GEICO.20 Oncologia 2, Istituto Oncologico Veneto, Padua, Italy. 21 Institut Catalá de Oncología de Hospitalet, Barcelona, Spain and GEICO.22 Obstetrics and Gynaecology, ASST Spedali Civili-Università degli Studi di Brescia, Brescia, Italy.23 Hospital Reina Sofía Córdoba, Spain and GEICO.24 S.C. Oncologia Medica, E.O. Ospedali Galliera, Genoa, Italy. 25 IRCCS Istituto Romagnolo per lo Studio dei Tumori (IRST) "Dino Amadori", Meldola, Italy,26 AOU Città della Salute e della Scienza - OIRM S. Anna Torino, Italy.27 AOU Policlinico Umberto I di Roma, Rome Italy.28 Istituto di Candiolo, FPO-IRCCS and University of Torino, Italy 29 Ospedale Infermi-Rimini, Italy. Italy.30 IRCCS Azienda Ospedaliero-universitaria di Bologna, Bologna, Italy.31 Azienda Unità Sanitaria Locale di Reggio Emilia IRCCS, Reggio Emilia, Italy.32 Amsterdam UMC - Amsterdam, Netherlands. 33 Universitätsspital Basel, USB, Switzerland and SAKK 34 NOGGO Study group and Department of Gynecology and Obstetrics, Technische Universität Dresden, Fetscherstr, Dresden, Germany; National Center for Tumor Diseases (NCT/UCC), Dresden, Germany: German Cancer Research Center (DKFZ), Heidelberg, Germany; Faculty of Medicine and University Hospital Carl Gustav Carus, Technische Universität Dresden, Dresden, Germany; Helmholtz-Zentrum Dresden - Rossendorf (HZDR), Dresden, Germany.35 Initia Oncologia, Valencia, Spain and GEICO. 36 Istituto Europeo di Oncologia, Milano, 37 Ospedale Civile, Sondrio, Italy. 38 IRCCS Ospedale Policlinico San Martino, Genova, Italy, 39 Ospedale Del Ponte, Varese, Italy. 40 Azienda Ospedaliera S. Croce e Carle, Cuneo, Italy. 41 Fondazione Poliambulanza, Brescia, Italy. 42 AOU Maggiore della Carità, Novara, Italy. 43 Policlinico Umberto I, Università di Roma "La Sapienza", Roma, Italy. 44 Nuovo Ospedale di Prato S. Stefano, Prato, Italy.45 Azienda Ospedaliera SS Antonio e Biagio e Cesare Arrigo, Alessandria, Italy. 46 Istituto di Candiolo, FPO-IRCCS, Candiolo, Italy.47 Ospedale Degli Infermi, Biella, Italy.48 Ospedale SS Trinità, Sora,

Italy.49 IRCCS Policlinico S. Matteo, Pavia, Italy.50 AO Ordine Mauriziano, Torino, Italy.51 Ospedale Guglielmo da Saliceto, Piacenza, Italy.52 Azienda Ospedaliero Universitaria Policlinico di Modena, Modena, Italy. 53 Ospedale di Faenza, Faenza, Italy. 54 Ospedale Umberto I, Lugo, Italy. 55 U.L.S.S. 13 Mirano, Dolo, Noale, Italy. 56 AO della Provincia di Lecco, Ospedale Alessandro Manzoni, Lecco, Italy. 57 Ospedale di Santa Chiara, Trento, Italy. 58 Ospedale Regionale Umberto Parini, Aosta, Italy.59 Ospedale Valduce, Como, Italy. 60 Istituto di Ricovero e Cura a Carattere Scientifico - Centro Regionale Oncologico Basilicata, Rionero in Vulture, Italy. 61 P.O. "A.Perrino" ASL Brindisi, Italy. 62 ASL VC Ospedale S. Andrea, Vercelli, Italy. 63 Sacro Cuore Don Calabria, Negrar, Italy. 64 Ospedale S. Vincenzo, Taormina, Italy. 65 ARNAS Civico, Palermo, Italy. 66 Università degli Studi di Napoli Federico II, Napoli, Italy. 67 Ospedale San Giuseppe – Azienda USL11, Empoli, Italy. 68 Hospital La Fe, Valencia, Spain. 69 MD Anderson Cancer Center, Madrid, Spain. 70 Hospital Son Espases - Palma de Mallorca, Spain.71 Hospital General Universitario de Valencia, Valencia, Spain.72 Consorcio Hospitalario Provincial de Castellon, Castelló de la Plana, Spain. 73 H.U. Arnau de Vilanova, Lleida, Spain. 74 Consorci Sanitari De Terrassa, Terrassa, Spain. 75 Althaia, Manresa, Spain. 76 Hospital Universitario J.M. Morales Meseguer, Murcia, Spain. 77 Hospital Lluis Alcanyis Xativa, Valencia, Spain. 78 Corporacion Sanitaria y Universitaria Parc Tauli, Sabadell, Spain. 79 Hospital Universitario 12 de Octubre, Madrid, Spain. 80 Hospital General Universitario de Elche, Alicante, Spain. 81 Hospital Clinico Universitario Virgen De La Arrixaca, Murcia, Spain. 82 Charite Universitaetsmedizin Berlin, Germany.83 Schwerpunktpraxis für Gynäkologische Onkologie, Fürstenwalde, Germany. 84 Universitaetsklinikum Jena, Jena, Germany. 85 University Medical Center Hamburg, Hamburg, Germany. 86 University Hospital Dresden, Germany. 87 Praxisklinik Krebsheilkunde für Frauen, Berlin, Germany. 88 Vivantes Netzwerk für Gesundheit GmbH, Berlin, Germany. 89 Universitäts – Frauenklinik, Tübingen, Germany. 90 Universitaetsklinikum SH Campus Kiel, Kiel, Germany. 91 Kliniken Essen Mitte Evang. Huyssens Stiftung, Essen, Germany. 92 Universitatsmedizin Greifswald, Greifswald, Germany. 93 Universitatsfrauenklinik Dusseldorf, Dusseldorf, Germany. 94 Klinikum Leverkusen gGmbH, Leverkusen, Germany. 95 UFK am Klinikum Suedstadt Rostock, Germany. 96 Studienzentrum Onkologie, Ravensburg, Germany. 97 Universitätsklinikum der Johannes-Gutenberg-Universität Mainz, Mainz, Germany. 98 Obstetrics and Gynaecology - Universitatsklinikum Wuerzburg, Wuerzburg, Germany. 99 University Hospital Aachen, Aachen, Germany.100 Worthingh Hospital, Worthing, UK.101 Velindre Cancer Center – Whitchurch Cardiff, UK.102 Royal United Hospital Bath – Bath, UK.103 CHU Dinant Godinne / UCL Namur, Yvoir, Belgium. 104 UZ Leuven, Leuven, Belgium. 105 CMSE Clinique et Maternité Sainte-Elisabeth, Namur, Belgium.106 AZ Maria Middelares, Gent, Belgium. 107 Kantonsspital Graubünden, Chur, Switzerland. 108 Kantonsspital Aarau, Switzerland. 109 Universitätsklinik für Frauenheilkunde, Universitätsklinik für Onkologische Medizin – Inselspital, Bern, Switzerland. 110 Luzerner Kantonsspital, Switzerland.111 Kantonsspital Olten, Switzerland. 112 Kantonsspital Frauenfeld, Switzerland. 113 Kantonsspital, Winterthur, Switzerland. 114 Kantonsspital, Münsterlingen, Switzerland. 115 Radium Hospitalet Oslo University Hospital, Oslo, Norway. 116 University Hospital of North Norway, Tromsoe, Norway. 117 Stavanger University Hospital, Stavanger, Norway. 118 Oulu University Hospital, Oulu, Finland. 119 Kuopio University Hospital, Kuopio, Finland. 120 Medizinische Universitat Graz, Graz, Austria. 121 Krankenhaus Der Barmherzigen Brueder, Graz, Austria. 122 Univ. Klinik Frauenheilkunde AKH, Wien, Austria. 123 Scottish Clinical Trials Research Unit, Edinburgh, Scotland, UK. 124 Grupo Español de Investigación en Cáncer de Ovario – GEICO, Madrid, Spain. 125 NOGGO e.V.Nord-Ostdeutsche Gesellschaft für Gynäkologische Onkologie (North Eastern German Society for gynaecological oncology), Berlin, Germany. 126 Swiss Group for Clinical Cancer Research (SAKK), Bern, Switzerland. 127 Nordic Society of Gynaecological Oncology – Clinical Trial Unit, Copenhagen, Denmark.
